# Coarse-Grained Molecular Simulation of Epidermal Growth Factor Receptor Protein Tyrosine Kinase Multi-Site Self-Phosphorylation

**DOI:** 10.1371/journal.pcbi.1003435

**Published:** 2014-01-16

**Authors:** John G. Koland

**Affiliations:** Departments of Pharmacology and Internal Medicine, University of Iowa, Carver College of Medicine, Iowa City, Iowa, United States of America; Tel Aviv University, Israel

## Abstract

Upon the ligand-dependent dimerization of the epidermal growth factor receptor (EGFR), the intrinsic protein tyrosine kinase (PTK) activity of one receptor monomer is activated, and the dimeric receptor undergoes self-phosphorylation at any of eight candidate phosphorylation sites (P-sites) in either of the two C-terminal (CT) domains. While the structures of the extracellular ligand binding and intracellular PTK domains are known, that of the ∼225-amino acid CT domain is not, presumably because it is disordered. Receptor phosphorylation on CT domain P-sites is critical in signaling because of the binding of specific signaling effector molecules to individual phosphorylated P-sites. To investigate how the combination of conventional substrate recognition and the unique topological factors involved in the CT domain self-phosphorylation reaction lead to selectivity in P-site phosphorylation, we performed coarse-grained molecular simulations of the P-site/catalytic site binding reactions that precede EGFR self-phosphorylation events. Our results indicate that self-phosphorylation of the dimeric EGFR, although generally believed to occur *in trans*, may well occur with a similar efficiency *in cis*, with the P-sites of both receptor monomers being phosphorylated to a similar extent. An exception was the case of the most kinase-proximal P-site-992, the catalytic site binding of which occurred exclusively *in cis* via an intramolecular reaction. We discovered that the *in cis* interaction of P-site-992 with the catalytic site was facilitated by a cleft between the N-terminal and C-terminal lobes of the PTK domain that allows the short CT domain sequence tethering P-site-992 to the PTK core to reach the catalytic site. Our work provides several new mechanistic insights into the EGFR self-phosphorylation reaction, and demonstrates the potential of coarse-grained molecular simulation approaches for investigating the complexities of self-phosphorylation in molecules such as EGFR (HER/ErbB) family receptors and growth factor receptor PTKs in general.

## Author Summary

The epidermal growth factor receptor (EGFR) is one of a large group of cell surface receptors that allow cells to respond to growth-stimulating signals in their environment. Upon sensing of growth factor, the EGFR is activated, which triggers a signaling cascade leading to the cell nucleus and ultimately initiating cell division. The first event following receptor activation is an intramolecular kinase reaction that results in the introduction of phosphate groups onto several specific amino acids (phosphorylation sites or P-sites) in the tail of the EGFR protein. Thus, the tail of the receptor undergoes self-phosphorylation, which involves conformational motions enabling the various P-sites to access the catalytic site. The structure of the tail of the receptor is unknown, and hence the mechanism of the self-phosphorylation reaction is not well understood. To investigate this mechanism, we generated a structural model of the EGFR protein and performed computer simulations of EGFR P-site/catalytic site binding reactions. These simulations indicated how the distribution of P-sites along the tail of the receptor and restrictions in molecular movements of the tail lead to selectivity in the phosphorylation of the different P-sites. Our simulations yielded unique insights into the mechanism of EGFR self-phosphorylation that have important biological implications.

## Introduction

Classical polypeptide growth factor receptors are intrinsic membrane proteins, each possessing a ligand binding domain in the N-terminal extracellular portion of the molecule, a protein tyrosine kinase (PTK) domain in the C-terminal intracellular portion, and a single transmembrane (TM) domain in the intervening sequence [1]. The intrinsic PTK activity of the intracellular domain is activated upon a ligand-dependent dimerization of receptor monomers, with activation resulting in the self-phosphorylation (autophosphorylation) of both monomers on multiple tyrosine residues. These tyrosine residue phosphorylation sites (P-sites) are typically located within a lengthy phosphorylation domain (CT domain) at the receptor C-terminus, but in some cases also within a intracellular juxtamembrane (JM) sequence joining the TM domain and PTK core or a “kinase insert” sequence in the PTK domain proper. Receptor self-phosphorylation on P-sites triggers the binding of various signaling effectors via their Src homology 2 (SH2) or phosphotyrosine binding (PTB) domains, which both bind phosphorylated tyrosine residues in specific amino acid sequence contexts. Thus, the phosphorylation of specific P-sites within the receptor CT domain or elsewhere results in the activation of specific downstream signaling pathways that can be mitogenic (inducing of cell division), anti-apoptotic (promoting of cell survival), or regulatory of metabolism. While the differing sequences of phosphorylated receptor P-sites results in a selectively in their recruitment and activation of downstream signaling effectors, it is not known what determines the efficiency with which individual P-sites become phosphorylated upon receptor activation. This is in part because the structures of the various receptor CT domains are unknown, perhaps reflective of their generally disordered nature, and because the dynamics of these domains have not been examined.

A case in point is that of the prototypical epidermal growth factor receptor (EGFR, also termed HER1) PTK, about which there exists extensive structural information [2]. Here, in terms of the presumed ligand-activated dimeric receptor form, several independent structures of receptor extracellular domain dimers (with bound growth factor molecules) and PTK domain dimers have been presented, in addition to a recent structural model of the interaction of the two TM and two intracellular JM domains in the receptor dimer [3]. The elegant and painstaking studies of the Kuriyan lab have led to a generally accepted model in which the latent PTK activity of the monomeric EGFR is activated in the dimeric receptor form by an asymmetric allosteric interaction between PTK domains, in which binding of the C-terminal lobe of one PTK domain (the “activator”) to the N-terminal lobe of the other (the “receiver”) results in the activation of the receiver but not the activator [4]. Thus, in this model, and assuming no higher-order oligomeric interactions occur, the CT domains of the individual EGFR molecules (where all direct P-sites are known to reside [5]) must be phosphorylated either via an intramolecular reaction (a *cis* mechanism) in the case of the receiver molecule or an intermolecular reaction (*in trans*) in the case of the activator molecule. As the roles of the activator and receiver PTK domains might be exchanged by a dissociation and ensuant reassociation of the kinases in the opposite orientation [4], it is formally possible that the CT domain of an individual receptor molecule is phosphorylated by both *cis* and *trans* mechanisms.

In contrast to the extensively characterized extracellular and PTK domain structures, no structure for the ∼225-amino acid CT domain of the EGFR is available, except for those of short CT domain sequences that are seen to be ordered in different crystal structures, with variations in the sequences ordered between different crystal forms making the biologic relevance of these CT domain structures unclear (cf. [6]). Of eight candidate P-sites located within the CT domain (see Table 1), a range of *in vitro* phosphorylation studies (employing purified EGFR protein) and live cell phosphorylation analyses have identified the various major and minor sites of phosphorylation (Table 2, reviewed in [7]). There indeed is selectivity in phosphorylation site usage, with P-sites including Tyr-1068, Tyr-1086, Tyr-1148 and Tyr-1173 (hereafter designated P-site-1068, P-site-1086, etc.) being consistently identified as major sites of phosphorylation, and P-site-1101 not seen to be significantly phosphorylated. While the effect of P-site sequence on phosphorylation efficiency has been investigated with synthetic P-site-derived peptide substrates [8], [9], the sequence specificity seen in such experiments does not recapitulate the pattern of selectively seen in the actual self-phosphorylation reaction. Thus, whereas a P-site-1086-derived peptide was the poorest peptide substrate among those examined (P-site-992, -1068, -1086, -1114, -1148 and -1173) in one study ([8], see Table 1), P-site-1086 has been characterized as a major site of EGFR self-phosphorylation *in vitro* and in live cells (see Table 2).

**Table 1:** EGFR CT domain P-site sequences.

P-site	Sequence*	Category**	*k*_cat_*/K*_M_***	*v*_phos_****
992	DADE**Y**LIPQ	Minor	74.9	8.4
1045	FLQR**Y**SSDP	Minor	-	-
1068	PVPE**Y**INQS	Major	65.4	3.3
1086	QNPV**Y**HNQP	Major	12.1	0.3
1101	RDPH**Y**QDPH	-	-	-
1114	GNPE**Y**LNTV	Minor	49.8	1.7
1148	DNPD**Y**QQDF	Major	71.2	2.8
1173	ENAE**Y**LRVA	Major	64.0	2.9

The sequences surrounding each of the eight tyrosine residues in the EGFR CT domain are shown, with the tyrosine identifying the P-site highlighted in bold.

Qualitative categorization based on the consensus of experiments characterizing major or minor sites of EGFR self-phosphorylation (see Table 2; -, phosphorylation not detected).

Steady state catalytic efficiencies *k*_cat_/*K*_M_ (in units of mM^−1^ min^−1^) characterizing the phosphorylation of synthetic peptide substrates containing the P-site sequences by the ligand-activated EGFR as determined by Fan *et al.* [8] (-, not determined).

Predicted relative initial velocities of P-site self-phosphorylation computed as *v*_phos_ = *k′*_intra_.(*k*_cat_*/K*_M_), where *k′*_intra_ is relative frequency of binding site interactions for each P-site (the sums for those in both receiver and activator molecules) in simulations performed in the absence of CT domain electrostatic interactions (see Fig. 9 and *Supporting Information*, Text S1).

**Table 2:** Relative levels of phosphorylation of individual EGFR P-sites.

	P-site								
	992	1045	1068	1086	1101	1114	1148	1173	Citation
*in vitro* study			+++				+++	+++	[57]
			+++*	+++			+++*	+++*	[58]
			+++*	++			+++*	+++*	[5]
	+	++*	+++*	+++*			+++*	+++*	[59]
cultured cell study			++				++	+++	[57]
	−	+	+++	+++		+++	+++	+++	[35]
	NA	+++	+++	NA	NA	NA	+++	+**	[44]
	+++	++	+	++			+	+++	[34]

A survey of studies of EGFR phosphorylation site usage indicating relative phosphorylation of individual P-sites on a scale from +, ++ to +++ (−, phosphorylation negligible or not resolvable; NA, not analyzed).

implied in the cited study.

greatly enhanced by HER2 expression.

For growth factor receptors in general, selectivity in self-phosphorylation is of major significance with regard to signaling outcome. Thus, various laboratories have shown that mutation of individual P-sites (typically with tyrosine to phenylalanine substitutions) in growth factor receptor PTKs can alter the downstream signaling effectors activated and the physiologic response (e.g. induction of proliferation or cellular transformation in the case of oncogenic growth factor receptors) [10]–[13]. The EGFR and the other EGFR (HER/ErbB) family receptors (HER2, HER3 and HER4) are particularly plastic with regard to their signaling activities, as in addition to homodimeric receptor complexes, various heterodimeric complexes (e.g. EGFR/HER3 dimers) are formed. This dramatically alters signaling outcome because of the variety of P-sites presented and the diversity of downstream signaling effectors recruited by such heterodimeric complexes [14]. The biologic significance of HER/ErbB family receptor signaling specificity is evinced by the ongoing development of cancer therapeutic agents that target individual HER/ErbB family receptors [15].

When considering the dimeric EGFR as a molecular unit, the self-phosphorylation reaction, whether occurring by *cis* or *trans* mechanisms, is effectively intramolecular nature. We reasoned that the determinants of P-site usage might, in addition to the usual elements of enzyme-substrate recognition, also include topologic/configurational factors that influence the access of individual P-sites to the active site. Thus, because multiple P-sites within the lengthy CT domain of the EGFR can be phosphorylated and one CT domain can indeed be multiply phosphorylated [16], there must be a large range of CT domain conformations sampled over the time course of self-phosphorylation (seconds to minutes). How such conformational sampling contributes to the efficiency with which individual P-sites are phosphorylated has not to our knowledge been examined in the case of any self-phosphorylating protein kinase. We describe herein the development of a computational coarse-grained simulation method for modeling the EGFR self-phosphorylation reaction and its application in evaluating how the combination of enzyme-substrate recognition elements and the sampling of CT domain conformations effects selectivity in P-site phosphorylation.

## Results

### A complete structural model of the active dimeric EGFR

**Figure 1:**
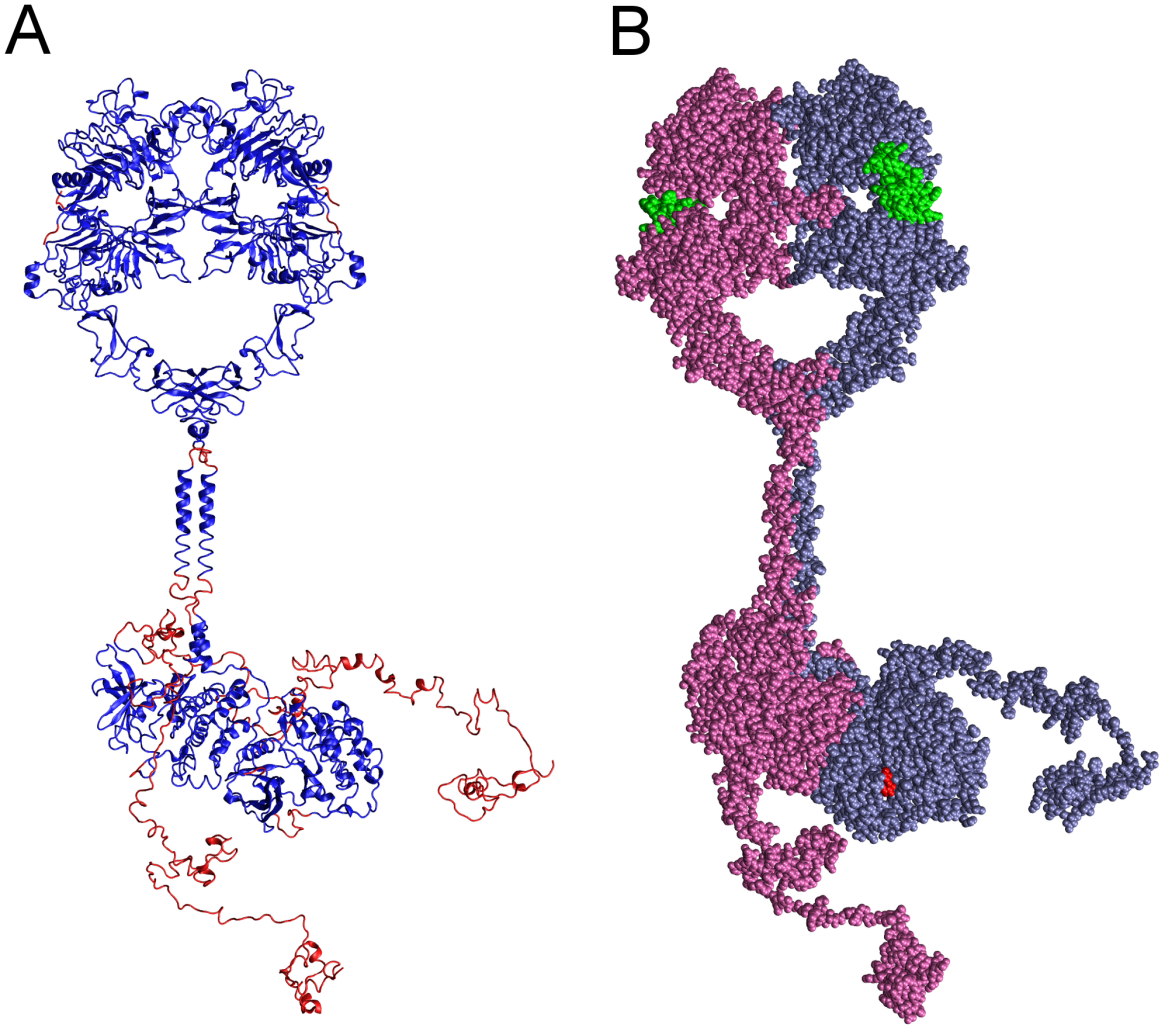
Complete structural model of the dimeric EGFR. Shown in two representations is the complete structural model of a dimer of EGFR molecules, each with a bound EGF molecule and bound AMPPNP^.^2Mg^2+^ substrate complex, generated herein. (A) Backbone conformation and secondary structure of the modeled EGFR polypeptides, with segments derived from published crystallographic structures colored blue and modeled segments colored red. (B) Van der Waals representation of the EGFR dimer, with the monomers having active (receiver) and inactive (activator) conformation kinases colored ice-blue and mauve, respectively, bound EGF molecules colored green, and bound AMPPNP^.^2Mg^2+^ substrate complexes colored red.

**Figure 2:**
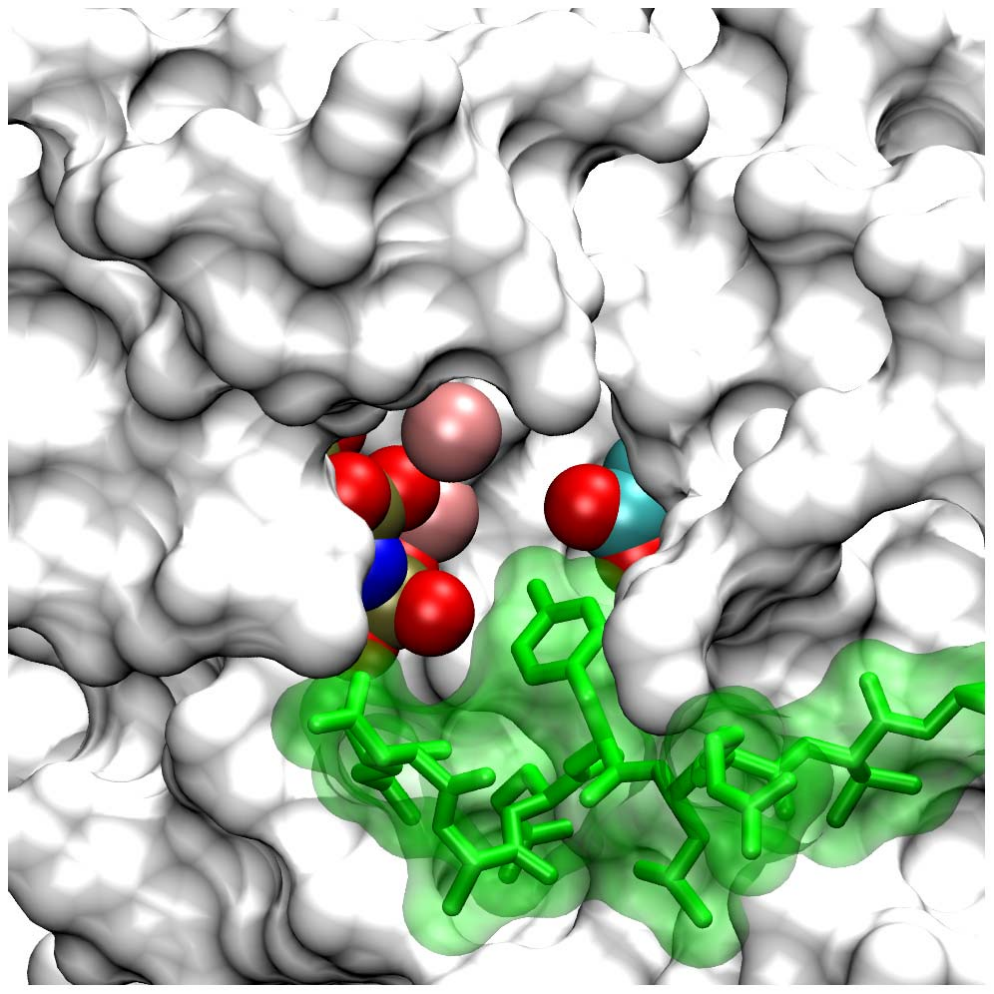
Active conformation PTK domain with bound nucleotide and peptide substrates in the complete EGFR model. Shown in an accessible surface representation are residues of the active conformation PTK domain (white) with its catalytic Asp-813 and bound AMPPNP^.^2Mg^2+^ substrate complex highlighted in CPK coloring. The docked nine-amino acid P-site-1173 peptide (sequence ENAEYLRVA) is shown in green. Note that the tyrosine hydroxyl of the peptide substrate is in close proximity to both the carboxyl group of Asp-813 and the γ-phosphate of the AMPPNP substrate analog. Similar models were generated with each of the eight distinct P-site peptides (see Table 1) docked in the active site.

For the purpose of simulating the EGFR self-phosphorylation reaction, we first created a complete structural model of the 1,186-amino acid EGFR holoreceptor (i.e. with full-length CT domain) in the active dimeric form (see Fig. 1). This structure was built up largely from the known structure of the EGFR extracellular domain with bound EGF in a 2∶2 “dimeric” complex (3NJP) [17] and an asymmetric PTK domain dimer structure formed from individual active (2GS6) and inactive (2GS7) conformation kinase structures [4] (see *Materials and Methods*). Sequences of unknown structure, including the two extracellular and two intracellular JM domain sequences of the dimeric molecule and the 219- and 227-amino acid CT domains of the receiver and activator, respectively, monomers, were structurally modeled using an in-house algorithm in the case of N- and C-terminal extensions and the Loopy algorithm [18] in the case of connecting segments. The active sites of each PTK domain in our model were occupied with an AMPPNP substrate analog and two Mg^2+^ cations (i.e. an AMPPNP^.^2Mg^2+^ substrate complex) (see Fig. 2).

Although there have been exciting recent applications of all-atom molecular dynamics methods in investigating various structural transitions associated with EGFR function (see *Discussion*), the large number of atoms in our dimeric EGFR structure and the presumed long time scale of receptor CT domain self-phosphorylation events precluded their simulation by all-atom explicit solvent molecular dynamics. Instead, we chose to simulate the EGFR self-phosphorylation reaction with a coarse-grained model of the dimeric EGFR and an implicit solvent Langevin dynamics method [19]. In this coarse-grained model and the associated energetic model, amino-acid residues were represented as pseudo-atoms centered upon their C_α_ atoms and physical interactions between pseudo-atoms were treated using the approach of Gō [20], much as implemented by others in studies of protein folding [21]–[25]. Thus, non-electrostatic interactions between non-bonded pseudo-atoms pairs were either short-range attractive for those representing native contacts (i.e. residues pairs interacting in EGFR elements of known structure) or strictly repulsive for all others (see *Materials and Methods*). This treatment of pseudo-atom interactions ensures that the native folds of structured protein domains remain stable in dynamic simulations. With the exception of residues in the CT domain P-sites, residues in modeled structural segments were not considered to make native contacts. By separately docking each of the eight nine-amino acid P-site sequences (see Table 1) in the active site of the active conformation PTK domain structure (see Fig. 2), we identified a set of “native” contacts characterizing each P-site/active site interaction, with identical contacts used to characterize the active site interactions of the two copies of each P-site present in the dimeric receptor and the potential *cis* or *trans* interaction of each P-site with the active site of either the receiver molecule (referred to hereon as the catalytic site) or the activator molecule (see *Materials and Methods*). We envisioned that the inclusion in the energetic model of short-range attractive potentials corresponding to the identified P-site/active site native contacts would allow the formation of stable P-site/catalytic site interactions in our simulations that would mimic those presumed necessary in the course of actual self-phosphorylation events.

### Dimeric EGFR models with randomized CT domain conformations

**Figure 3:**
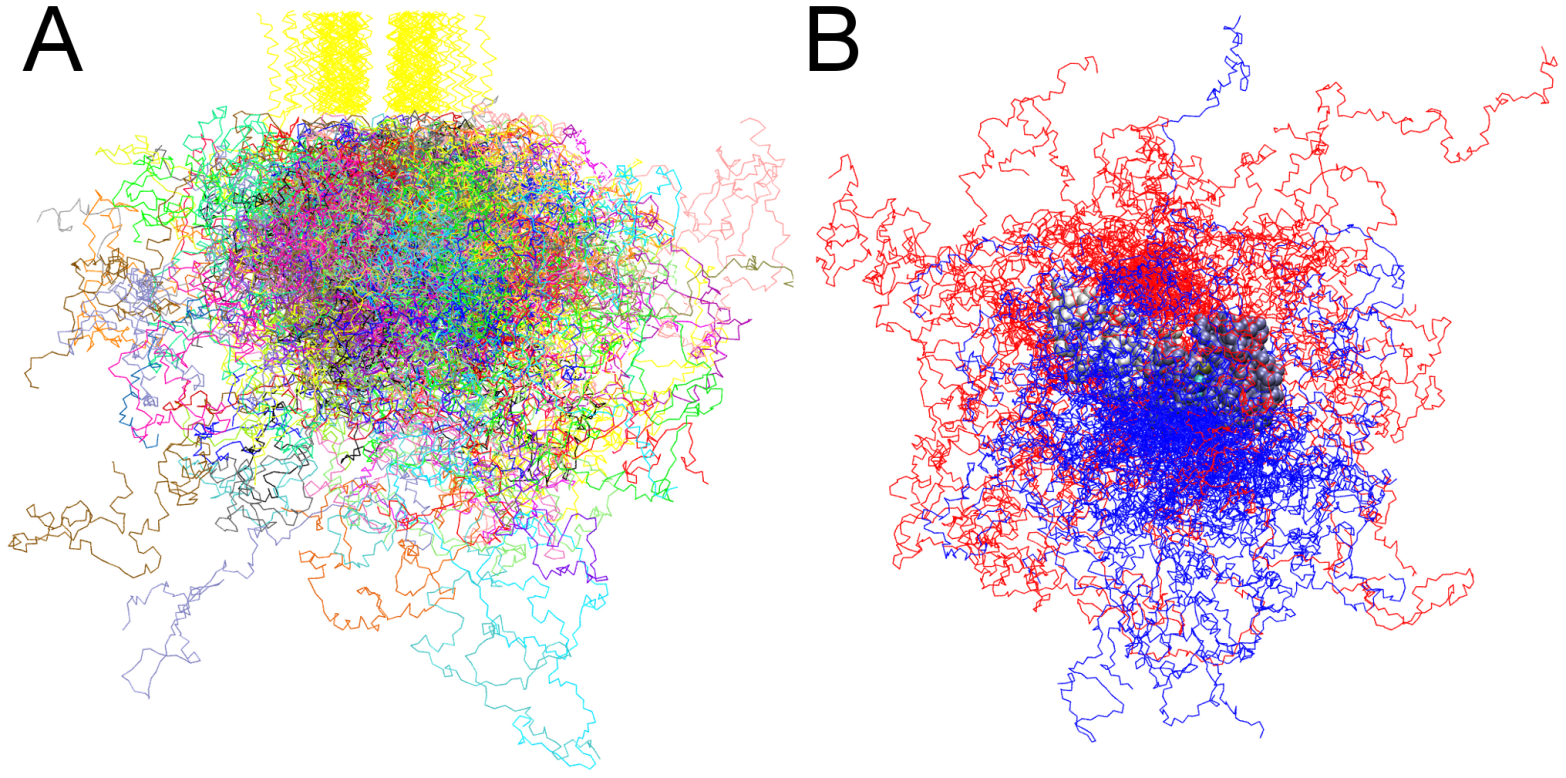
Conformation of the CT domains in randomized dimeric EGFR structural models. (A) Rendering of fifty randomized structures of the coarse-grained EGFR model showing the backbone conformation of the intracellular (in a different color for each structure) and TM (all in yellow) domains, with the TM domains of each structure together aligned. (B) Graphic of the same structures with the active conformation kinase domain of the receiver molecule in each aligned, the CT domains of the receiver (residues 968 to 1186) and activator (residues 960 to 1186) molecules colored in blue and red, respectively, and pseudo-atoms of the receiver (residues 679 to 967) and activator (residues 679 to 959) kinase domains depicted as ice-blue and white beads, respectively.

A principal goal of our study was to assess the relatively frequency with which each of the sixteen P-site elements in the active dimeric EGFR structure interacted with the catalytic site. We reasoned that this might be dependent upon the initial conformations of the CT domains in our model. To avoid biasing our results by use of a single EGFR model with CT domains of arbitrary conformation, we first generated a large set of dimeric EGFR structures with randomized CT domain conformations. This was done by removing the CT domains from our initial EGFR structural model and repeating the CT domain modeling (see *Materials and Methods*), to generate a total of five EGFR models with distinct randomly generated CT domain conformations. These five models were then used as initial structures in simulations of 10 µsec duration each, such that by sampling structures at 0.1 µsec intervals a total of five hundred independent EGFR structures with “randomized” CT domain conformation were obtained. In the energetic model used for these simulations, we removed all short-range attractive potential terms involving P-site residues, which precluded the occurrence of any stable P-site/active site interactions. The apparent randomization of CT domain conformations in the generated structures is exemplified in Fig. 3. An analysis indicated that the diversity of structures sampled from these trajectories of EGFR motion was sufficient to obviate any potential influence of the choice of initial structures upon the frequencies of P-site binding events in subsequent simulations (see *Supporting Information*, Fig. S1).

### Relative frequencies of catalytic site binding by individual EGFR CT domain P-sites

**Figure 4:**
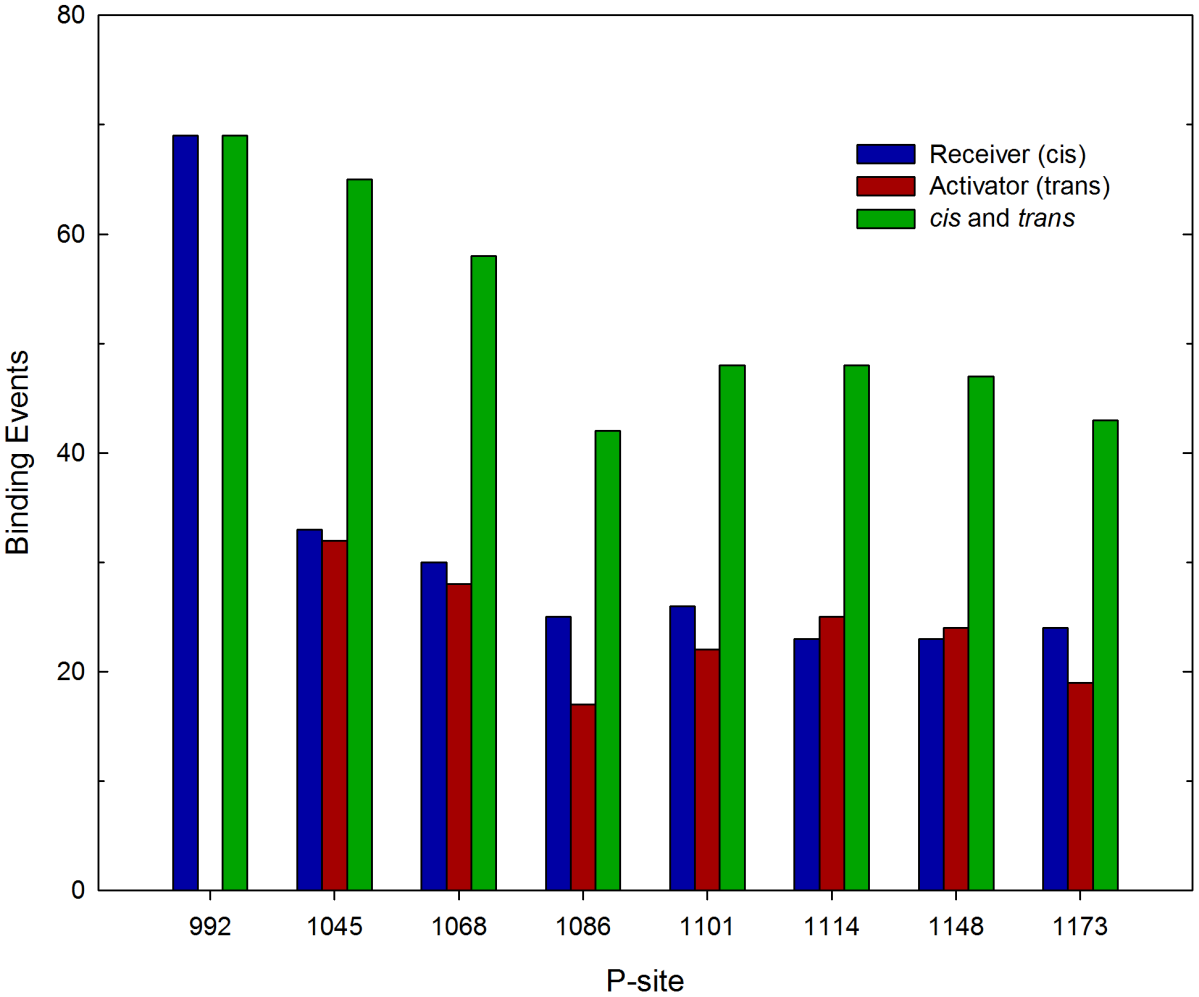
Frequency of P-site/catalytic site interactions in iterative simulations of the EGFR self-phosphorylation event. The frequencies of stable interactions of individual P-sites with the catalytic site were tabulated following 420 total molecular simulations, each performed using an initial structure randomly selected from a set of five hundred dimeric EGFR structures with randomized CT domain conformations (see Fig. 3). Interactions of individual P-sites in the CT domains of the receiver and activator molecules are separately tabulated, as are the sums of interactions of the P-sites in both chains (*cis* and *trans*). Note there was a general trend of reduced frequency of P-site/catalytic site binding events with increasing residue number, with the kinase-proximal site P-site-992 showing the most frequent interactions. Also, the catalytic site interactions of P-site-992 occurred exclusively *in cis* versus *in trans*.

Given this large set of dimeric EGFR structures with randomized CT domain conformations, we performed repeated simulations to assess the relative frequency with which each of the sixteen CT domain P-sites interacted with the catalytic site in a manner consistent with the occurrence of a self-phosphorylation reaction. Thus, in successive simulations, an initial EGFR structure was randomly selected from the set of five hundred, and the motion of the assemblage propagated until a stable P-site/catalytic site interaction occurred (see *Materials and Methods*), in which case the simulation was terminated, the elapsed time and the identity of P-site encountering the catalytic site recorded, and the process repeated. Running these iterative simulations on five 48-core servers in parallel for 58 days, we accumulated a histogram of the number of catalytic site binding events for each P-site, representing a total of 420 P-site/catalytic site interactions simulated (see Fig. 4). The average simulated time for a binding event was 0.46 µsec, over a total simulated time of 193 µsec. In several of the more protracted simulations, one or more P-site residues bound to the alternate active site of the activator molecule (events assumed to be nonproductive in terms of P-site phosphorylation), which appeared to delay the interaction of P-sites in the same CT domain with the true catalytic site (see *Supporting Information*, Videos S1, S2, S3 and Fig. S2). Thus, to some extent, the activator molecule competed with the catalytic receiver molecule in P-site binding, a phenomenon potentially relevant in the context of heterodimeric receptors containing the kinase-impaired HER3 protein (see *Discussion*).

Regarding the relatively frequency with which different P-sites interacted with the catalytic site, some general observations can be made. First, the summed frequencies of binding for all P-sites of the receiver molecule (*cis* binding events, *n = *253, where *n* is the number of binding events) was higher (*p*<0.0001) than that for P-sites of the activator molecule (*trans* binding events, *n* = 167). Interestingly, the frequency of binding for individual P-sites of the receiver molecule (*cis* binding events) was highest (*p*<0.05) for the site most proximal in sequence to the PTK domain (i.e. P-site-992, *n* = 69) and lower for more distal sites (e.g. P-site-1114 and -1148 each had 23 *cis* binding events each). With two exceptions, the frequencies of binding for differing P-sites of the activator molecule (*trans* binding events) mirrored those of the receiver molecule (*cis* binding events), ranging from 17 to 32 events. The exceptions here were that P-site-1086 of the activator molecule had a lower number of binding encounters (*n* = 17) than that of the receiver molecule (*n* = 25) (although this was not statistically significant) and that P-site-992 of the activator molecule had no encounters during the simulations. The discrepancy between the frequencies of *cis* versus *trans* binding for P-site-992 was responsible for the overall bias in favor of *cis* binding events, as when P-site-992 was ignored in the analysis, the numbers for *cis* (*n* = 184) versus *trans* (*n* = 167) binding events were not statistically different. Also, because the unusually high number of P-site-992 *cis* binding events was negated by a lack of P-site-992 *trans* binding events, the summed frequencies of *cis* and *trans* binding events for each P-site (see Fig. 4), which should be related to their relative propensities for phosphorylation as determined experimentally, were less variable overall ranging from *n* = 42 for P-site-1086 to *n* = 69 for P-site-992. The relatively low frequency of simulated P-site-1086 binding events is consistent with a previous steady state kinetics investigation of the phosphorylation by the EGFR of synthetic peptide substrates representing individual EGFR P-sites, wherein a P-site-1086-based peptide was found to be the poorest substrate among those examined [8] (see Table 1). However, P-site-1086 has been identified in various biochemical experiments as either a major or minor site of EGFR self-phosphorylation (see Table 2 and *Discussion*).

**Figure 5:**
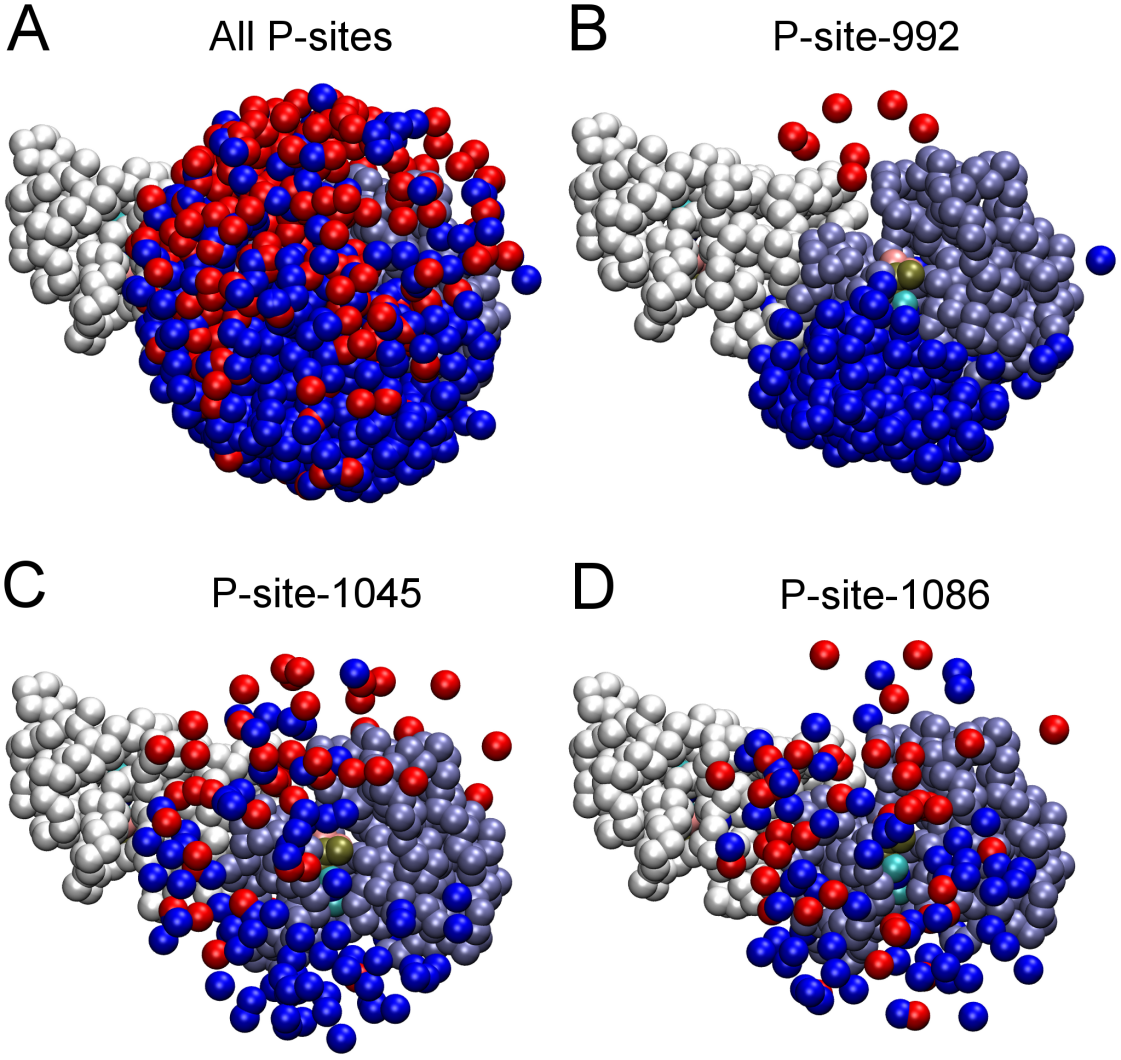
Proximity of P-site tyrosine residues to the catalytic site in randomized EGFR structural models. Renderings of the five hundred randomized structures used as initial structures in simulations after alignment of their receiver kinase domains are shown. Pseudo-atoms of one receiver (residues 679 to 967) and one activator (residues 679 to 959) kinase domain are depicted as ice-blue and white beads, respectively, and those representing P-site tyrosines in the CT domains of receiver and activator molecules depicted as blue and red beads, respectively. (A) Shown are those pseudo-atoms of all P-site tyrosine residues within 40 Å of the γ-phosphate of the AMPPMP substrate (tan bead) bound in the catalytic site of the receiver molecule. (B–D) Similar renderings but with only the tyrosines of P-site-992 (B), -1045 (C) or -1086 (D) shown. Note an apparent bias in the access of P-site-922 of the receiver molecule to the catalytic site. The number of P-sites of each variety in the vicinity of the catalytic site is quantified in Fig. 6.

**Figure 6:**
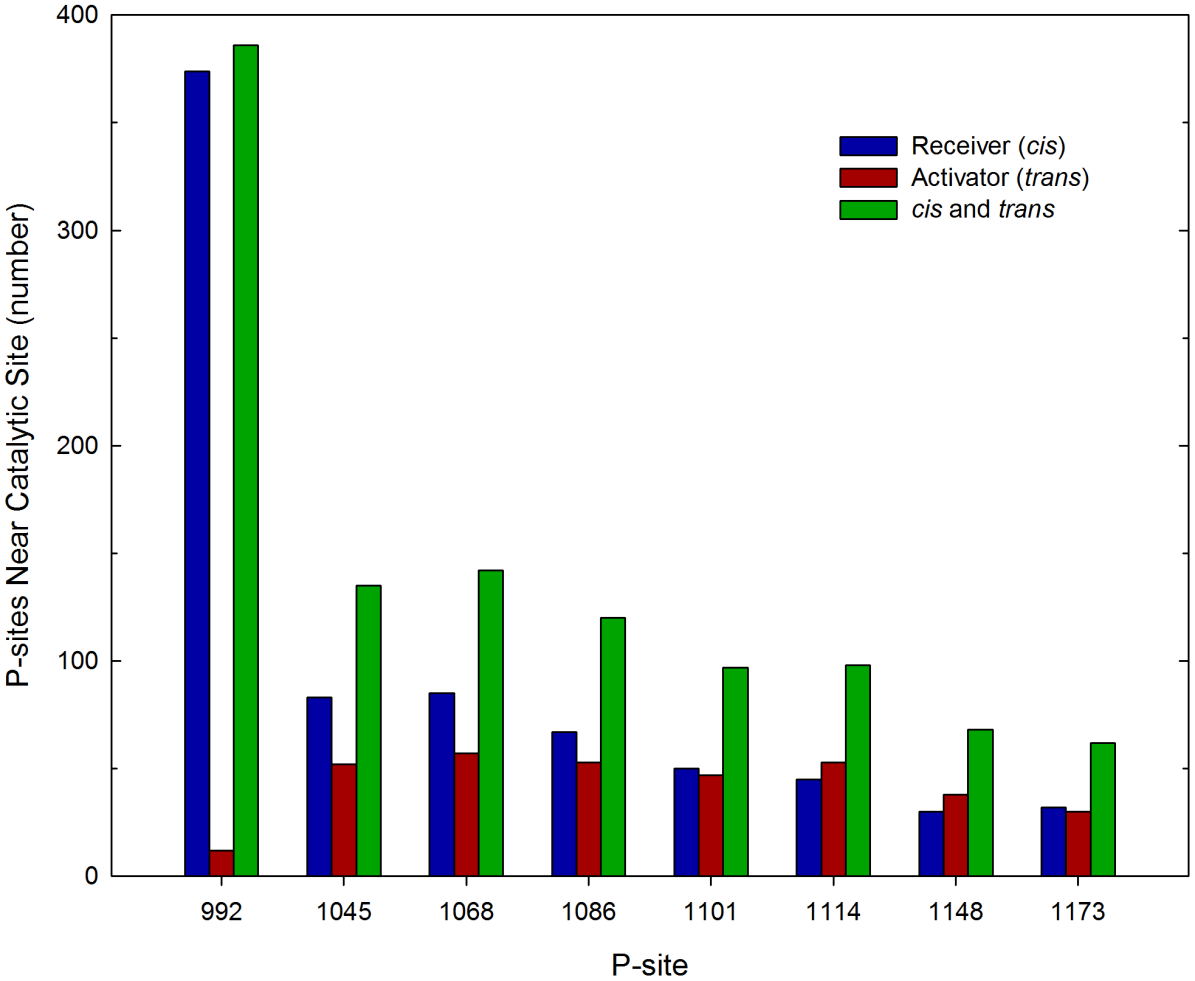
Number of P-sites in proximity of the catalytic site in randomized EGFR structural models. The set of five hundred dimeric EGFR structures with randomized CT domain conformations was analyzed to determine the number of structures that had a given P-site tyrosine residue within 40 Å of the γ-phosphate of the AMPPMP substrate bound in the catalytic site. The kinase-proximal site P-site-992 of the receiver molecule was most often and that of the activator molecule least often near the catalytic site, consistent with the bias for *cis* versus *trans* P-site-992 binding events in simulations (cf. Fig. 4). This *cis-trans* bias, as well as the number in proximity of the catalytic site, was markedly reduced for P-sites more distal to the kinase core.

The relative frequency of catalytic site interactions for an individual P-site might be dependent upon topologic factors (e.g. whether its access to the catalytic site is dependent upon its presence in the CT domain of the receiver versus the activator molecule or its location in the CT domain sequence being more proximal versus distal to the PTK core). Thus, some P-sites in trajectories of EGFR molecular motion might make more frequent excursions near the catalytic site, independent of conventional enzyme/substrate recognition phenomena, and for this reason be more efficiently phosphorylated. To assess how topologic factors might alter the frequency of catalytic site interactions of individual P-sites, we examined the set of five hundred EGFR structures taken from five simulated trajectories of EGFR motion (derived using a model without P-site/catalytic site native contacts) to ascertain the frequency with which individual P-sites were in proximity of the catalytic site (see *Materials and Methods*). The graphics of Fig. 5 show those P-sites in five hundred EGFR structures that were located within a radius of 40 Å from the γ-phosphate of the AMPPMP substrate in the catalytic site, the numbers of which are quantified in Fig. 6. Focusing on P-site-922 (Figs. 5B and 6), it appears that topologic factors potentially had a dramatic influence upon the relative frequency of catalytic site interactions for some P-sites, with P-site-992 of the receiver molecule found much more frequently within the vicinity of the catalytic site (a *cis* interaction) than was P-site-992 of the activator molecule (a *trans* interaction). However, the relative frequencies of catalytic site interactions of individual P-sites did not seem to be entirely determined by topologic factors. Thus, P-site-1086 had the lowest frequency of catalytic site binding events (see Fig. 4), although its frequency in the vicinity of the catalytic site was higher than that of four other P-sites (Fig. 6).

**Figure 7:**
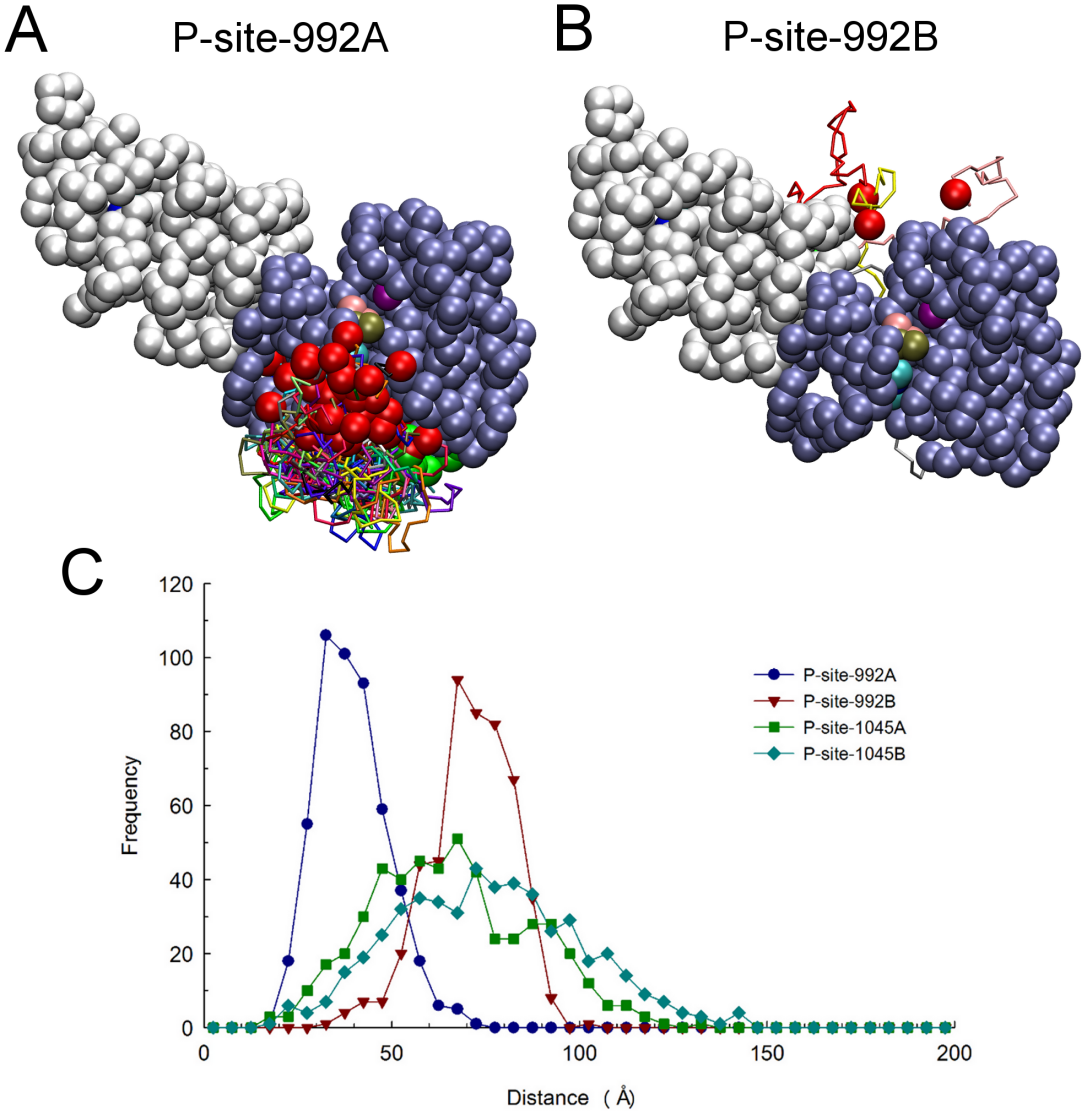
Relative catalytic site access of P-site-992 of the receiver and activator molecules. Structures were examined among a set of 650 selected at 100 µsec molecular trajectories of EGFR dimer motion (from simulations in which P-site/catalytic site residues pairs were not considered to form native contacts), to identify subsets in which P-site Tyr-992 of the receiver and activator molecules were within 30 Å of catalytic Asp-813 of the receiver. Compared here are the identified catalytic site excursions of P-site-922 of the receiver (A) and activator (B) molecules, after alignment of receiver PTK domains (residues 679 to 967) in the identified structures. One receiver and one activator PTK domain are shown as ice-blue and white beads, respectively, with the catalytic Asp-813 as a purple bead and the bound nucleotide substrate complex as beads with CPK coloring. The backbones of the partial CT domains of receiver (residues 968 to 991) and activator (residues 960 to 991) molecules are shown in different colors, with residue 967 or 959 at the origin of these domains and their target Tyr-992 depicted as green and red beads, respectively. Comparison of the two panels indicates that P-site-992 of the receiver made much more frequent excursions near the catalytic site. (C) Distances from the pseudo-atoms representing tyrosine residues of the indicated P-sites to the γ-phosphate of the AMPPMP substrate bound in the catalytic site were quantified for each of the five hundred randomized structural models used as initial structures for simulations and the distance distributions plotted. Note that while there was an obvious bias in the catalytic site proximities of the receiver P-site-992 (P-site-992A) versus that of the activator (P-site-992B), this bias was markedly reduced for P-sites more distal to the kinase core (e.g. P-site-1045A versus -1045B).

Regarding the discrepancy in observed binding events for P-site-992 of the receiver (69 of 420 total events) versus activator (0 of 420 total events) molecule, we considered that it might be physically impossible for P-site 992 to make an *in trans* interaction with the catalytic site. This issue was explored by examining the conformations of the two CT domains in each of 650 structures sampled from trajectories of EGFR motion to identify those structures in which P-site Tyr-992 of either the receiver (Fig. 7A) or activator (Fig. 7B) molecule were within 30 Å of catalytic Asp-813 of the receiver. A marked discrepancy in excursions near the catalytic site was evident. The range of P-site-992/catalytic site distances sampled during long trajectories of EGFR structural randomization is shown in Fig. 7C, which indicates that P-site-992 of the activator molecule (P-site-992B) was rarely in close proximity to the catalytic site. Thus, it appeared possible that the 24-amino acid sequence connecting Tyr-992 to the PTK domain was not of sufficient length to allow its interaction *in trans* with the catalytic site. While such an interaction might be physically possible (indeed a small number of such *trans* P-site-992/catalytic site encounters were observed in simulations with no CT domain electrostatic interactions as described below), it seemed that the lack of occurrences of *in trans* P-site-992/catalytic site binding events in our simulations (see Fig. 4) was due to the topologic arrangement of the kinase-proximal CT domains in the asymmetric dimer structure (see *discussion*).

**Figure 8:**
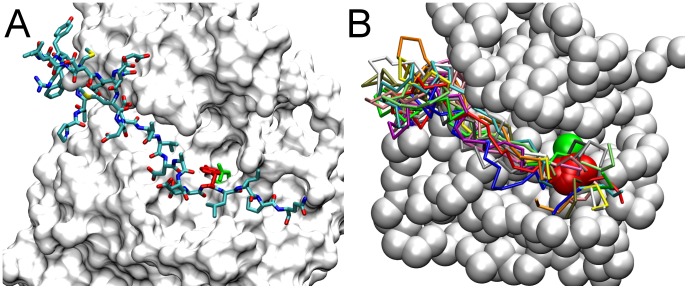
A substrate binding cleft facilitates the *cis* interaction of the kinase-proximal P-site-992 with the catalytic site. (A) After docking the P-site-992 peptide (residues 988 to 996) in the catalytic site, the structure of the CT domain sequence between it and the kinase core (residues 968 to 987) was modeled with the program Loopy [18] (see *Materials and Methods*). The resulting all-atom structure is shown with the solvent accessible surface of PTK domain residues 669 to 967 colored white, modeled CT domain residues 968 to 996 in CPK coloring, and the target Tyr-992 and catalytic Asp-813 residues highlighted in red and green, respectively. The modeled CT domain sequence was found to thread through a cleft between the N- and C-terminal lobes of the PTK domain, suggesting that the presence of this cleft facilitates access *in cis* of the kinase-proximal P-site-992 to the catalytic site. (B) In simulations of P-site/catalytic site binding events (Fig. 4), the most frequent events (69 of 420) were *cis* interactions of P-site-992 of the receiver molecule with the catalytic site. Shown here are the final structures from fourteen simulations randomly chosen from those ending with a *cis* P-site-992 binding event, after alignment of the receiver PTK domain pseudo-atoms 679 to 967 (those of one PTK domain shown as chalk-white beads). The backbone conformations of each of the fourteen CT domain sequences (residues 968 to 996) are shown in different colors, with the target Tyr-992 and catalytic Asp-813 depicted as red and green beads, respectively.

In contrast to the apparent inability of P-site-992 of the activator molecule to interact *in trans* with the catalytic site, P-site-992 of the receiver molecule had the most catalytic site binding events of any P-site. In our randomization of the EGFR CT domain structure, P-site-992 of the receiver molecule (P-site-992A) made very frequent excursions near the catalytic site (see Figs. 7A and C). Considering again that the propensity for this *cis* P-site-992/catalytic site interaction might reflect topological factors, especially its location in sequence being most proximal to the kinase core of the receiver molecule, we modeled the conformation of the CT domain segment connecting an active site-docked P-site-992 to the kinase core (see Fig. 8A). This modeling indicated that the connecting sequence was of a seemingly ideal length to allow a *cis* interaction of P-site-992 with the catalytic site. To our surprise, we observed that the stretch of residues 968 to 987 in the connecting CT domain sequence was threaded through a cleft in the PTK domain structure formed by an opening between the N- and C-terminal lobes of the kinase. To explore the possibility that this cleft served to enhance the access *in cis* of P-site-922 to the catalytic site, we examined a set of structures randomly selected from those representing P-site-922 binding events that occurred in our simulations (see Fig. 8B). Likely because of its limited length, the 24-residue CT domain sequence joining P-site Tyr-992 to the kinase core was again found threaded through the same peptide substrate binding cleft identified in our modeled structures. We also noted that the docking of P-site-992 in the active site was attended by favorable electrostatic interactions between Asp-988 and Glu-991 of the P-site sequence and Arg-779 and Arg-817, respectively, in the active site. The favorability of negative charges in the *N*-1 and *N*-4 positions of P-site sequences in terms of their activity as PTK substrates has been previously noted [26]. Thus, it appeared possible that the high frequency of P-site-992 interactions with the catalytic site *in cis* reflected both its favorable positioning in the CT domain sequence and the favorable charge interactions of P-site-992 residues with the catalytic site. Interestingly, among the three other HER/ErbB family receptors, HER2 and HER4 each possess a candidate P-site homologous to P-site-922 with acidic residues in the *N*-1 and *N*-4 positions, despite their CT domains being otherwise highly divergent in sequence (see Table 3 and *Discussion*).

**Table 3:** Conservation of the kinase-proximal CT domain sequences of HER/ErbB family receptors.

	* : . ::: :: :* : * : :
EGFR	968-PT**D**SNFYRALM**DEED**M**DD**VV**D**A**DEY**LIPQQGFFSSPSTSR
HER2	977-PL**D**STFYRSLL**EDDD**MG**D**LV**D**A**EEY**LVPQQGFFCPDPAPG
HER3	971-P**E**PHGLTNKKL**EE**V**E**L**E**P**E**L**D**L**D**L**D**L**E**A**EED**NLATTTLGS
HER4	973-PN**D**SKFFQNLL**DEED**L**ED**MM**D**A**EEY**LVPQAFNIPPPIYTS

A multiple sequence alignment (Clustal Omega algorithm, http://www.ebi.ac.uk/Tools/msa/clustalo/) of the four human HER/ErbB family receptor sequences was performed, with sequence conservation in the kinase-proximal CT domain shown here. The high conservation of the HER/ErbB PTK domain sequences extended at least twenty-five residues into the CT domain, and thus included P-site-992 of the EGFR, with homologous P-sites seen in the cases of HER2 and HER4 but not in the case of the kinase-impaired HER3. EGFR Tyr-992 and its homologs in HER2 and HER4 and acidic residues are shown in bold lettering. Numbering of the first CT domain residue in each receptor is that of the mature receptor form, i.e. with the predicted signal peptide removed.

fully conserved residue.

:strongly similar residue.

.weakly similar residue.

### Role of electrostatic interactions in P-site/catalytic site binding

**Figure 9:**
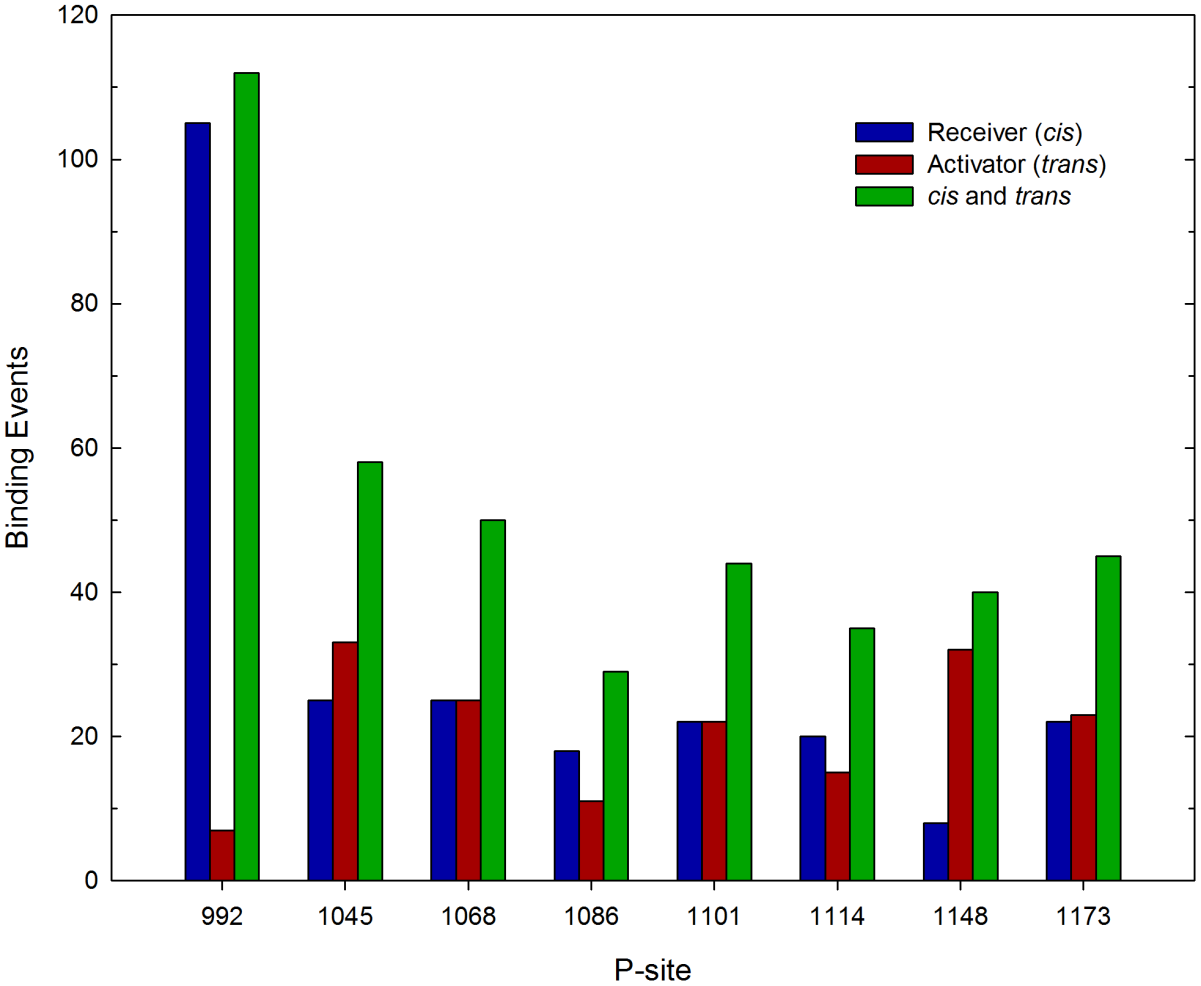
Effect of CT domain electrostatic interactions on the frequency of P-site/catalytic site binding events. The frequency of stable interactions of individual P-sites with the catalytic site was tabulated following 413 total simulations performed with a model in which all CT domain charges were eliminated. Interactions with the catalytic site of individual P-sites in the CT domains of the receiver and activator molecules are separately tabulated, as are the sum of interactions of the P-sites in both chains (*cis* and *trans*). Compared to what was seen in simulations with CT domain charges included (cf. Fig. 4), P-site-922 had a much enhanced tendency for *in cis* catalytic site interactions.

To examine the role that electrostatic interactions might play in facilitating or inhibiting the interaction of individual P-sites with the catalytic site, we performed further simulations using an EGFR model in which the charges on all pseudo-atoms representing CT domain residues (residues 968–1186 and 960–1186 of the receiver and activator molecules, respectively) were set to zero (see Fig. 9). This EGFR model did however include native contacts and associated short-range attractive potentials to stabilize P-site/catalytic site interactions. In agreement with our first simulations with a model including CT domain electrostatic interactions (compare Fig. 4), the P-site most proximal to the kinase core, P-site-992, interacted significantly more frequently (*p*<0.05) with the active site than did other more distal P-sites, and this site interacted almost exclusively *in cis*. This indicated that the high frequency of P-site-992 interactions observed in our first simulations was largely attributed to topological factors that much facilitated the *in cis* interaction of P-site-992 with the active site, including the short length of the sequence between the catalytic domain and P-site-992 and the substrate binding cleft we identified above (see Fig. 8). Hence, although the catalytic site interaction of P-site-992 involved residues of complementary charge (see above), the inclusion of electrostatic interactions in our initial simulations (compare Figs. 4 and 9) if anything negated the positive influence that topologic factors imparted upon the frequency of P-site-992 catalytic site binding. This possibly reflected an unfavorable electrostatic interaction of the CT domain sequence elements (residues 968 to 987, see Table 3) with the peptide substrate binding cleft that otherwise facilitates P-site-992 binding *in cis* (see Fig. 8 and *Discussion*). The relatively low frequency of P-site-1086 binding events seen in our original simulations with CT domain electrostatic interactions included was recapitulated in those in which CT domain charges were removed, suggesting that for this P-site electrostatic interactions between the CT domain and the kinase core did not greatly affect its potential interactions with the catalytic site. An as yet unexplained observation was a statistically significant bias (*p*<0.05) in favor of *trans* (*n* = 32) versus *cis* (*n* = 8) binding events for P-site-1148 in simulations with no CT domain electrostatic interactions that was not evident in our initial simulations.

### Modeling P-site selectivity with accumulated EGFR phosphorylation

The simulations described above were designed to model the first P-site binding event occurring upon receptor activation, such as would be reflected in an analysis of EGFR self-phosphorylation under initial velocity conditions. Given the possibility that multiple CT domain phosphorylations (phosphorylation stoichiometries exceeding one) might occur *in vivo*, we considered that the pattern of P-site phosphorylation subsequent to an initial phosphorylation event might differ significantly from that of the first. Hence, we simulated P-site binding events subsequent to a first event that resulted in the phosphorylation of a single P-site, using the initial phosphorylation of P-site-992A of the receiver monomer (the most frequently bound P-site) as an example. The simulation method applied above was modified so that iterative simulations were initiated with structures randomly chosen from a set representing 59 different P-site-992A binding events that occurred in our initial P-site binding simulations (see Fig. 8B). Also, in these simulations a formal negative charge was given to the pseudo-atom representing Tyr-992 of the receiver molecule to mimic its phosphorylation and all native contacts and corresponding short-range attractive potentials were eliminated for the nine residues of P-site-992A to eliminate its binding interaction with the catalytic site. Thus, each simulation began with the release of “phosphorylated” P-site-992A from the catalytic site, and continued until a second P-site was bound.

**Figure 10:**
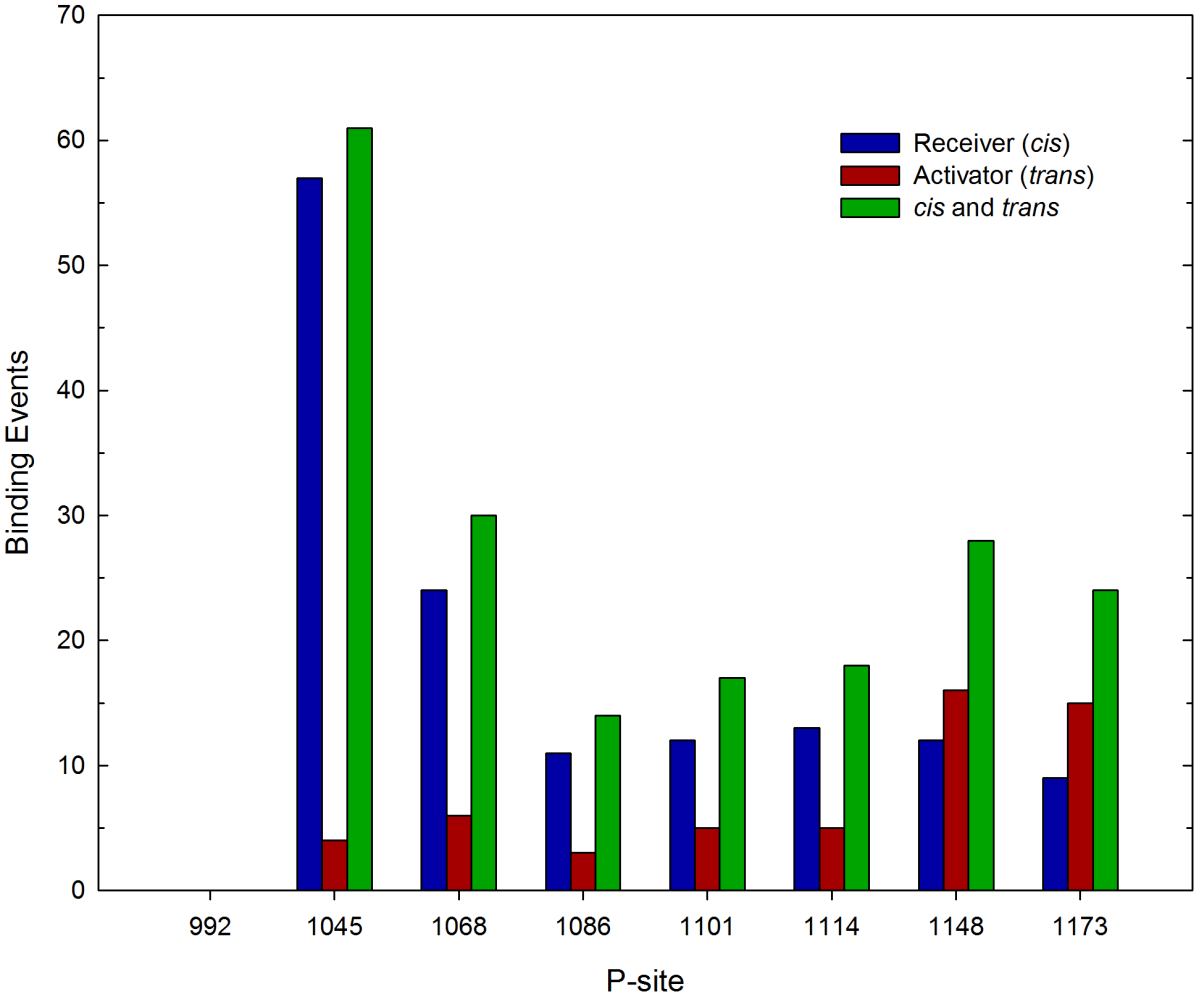
Second P-site binding event following an initial binding of P-site-992A. Repeated simulations were initiated with structures representing the dimeric EGFR with P-site-992A of the receiver monomer bound in the catalytic site (see Fig. 8B), and with the nascent phosphorylation of P-site-992A mimicked by the introduction of a negative charge and the removal of its P-site/active site interaction potentials in the simulation model (see text). Shown here are the frequencies with which individual P-sites underwent a subsequent P-site binding event over a course of 192 total simulations. A notable bias in favor of cis (Receiver, *n* = 138) versus trans (Activator, *n* = 54) binding events was seen, with the sites closest in sequence to P-site-992 of the receiver molecule binding most frequently.

In the results of these second P-site binding event simulations (see Fig. 10), we first observed there was a significant bias in favor of *cis* (Receiver, *n* = 138) versus *trans* (Activator, *n* = 54) binding events (*p*<0.05). This is noteworthy in that in our earlier simulations (Fig. 4) there was no significant *cis* versus *trans* bias except in the case of P-site-992, whose binding *in cis* was precluded in second binding event simulations by the mimicking of its phosphorylation in the receiver molecule. Here, a *cis* versus *trans* bias was seen in the case of the more kinase proximal P-sites (P-sites-1045 and -1068, *p*<0.05) and was most obvious in the case of P-site-1045, with the binding *in cis* of P-site-1045 being the most frequent binding event overall (*n* = 57) and its binding *in trans* occurring much less frequently (*n* = 4). Our simulation results, appeared to reflect a potential processivity in EGFR phosphorylation, i.e. the phosphorylation of one P-site (here P-site-992 of the receiver) greatly enhanced the likelihood that a neighboring P-site (P-site-1045 of the receiver) would be bound to the catalytic site and in turn phosphorylated (see *Discussion*). An enhanced propensity for the sequential binding of neighboring P-sites was also suggested by an analysis of non-productive P-site binding events (see *Supporting Information*, Fig. S2).

## Discussion

The mechanism and P-site selectivity of EGFR PTK multi-site self-phosphorylation was investigated herein through molecular simulations employing a coarse-grained model of the active dimeric EGFR. The all-atom structural model from which this coarse-grained model was derived was unique in representing a dimer of EGFR molecules with bound nucleotide substrates and full-length CT domains, with the 3-D structure of the latter not having been experimentally determined. Whereas several all-atom molecular dynamics and targeted molecular dynamics simulations of the EGFR kinase domain have been published, most were limited to investigating the dynamics of the kinase core either in isolation or in a dimeric form, and focused upon the transition between its inactive and active conformations [27]–[30] or the impact of oncogenic mutations on kinase inhibitor binding [31]. The structural models examined in these investigations included at most only a small segment of the CT domain sequence and shed little light upon the mechanism of self-phosphorylation. The all-atom molecular dynamics study of Mustafa *et al.* [32] investigated a kinase domain model with a significant segment (residues 951 to 994) of the CT domain included. The structure of the CT domain segment in this model was based on two partial CT domain structures seen to be ordered in different crystal structures of the kinase, although the connectivity of these structured CT domain segments with a single kinase molecule in the unit cell appears subject to uncertainty [6]. The study examined the coordinated movements of the CT and JM domains over a period of ∼10 nsec, and although the structure simulated included P-site-992, there was no movement of the CT domain on this time scale that approximated a self-phosphorylation event. We envisioned that simulating multiple self-phosphorylation reactions involving P-sites distributed throughout the full-length CT domains of the dimeric EGFR would require a much longer total time of simulation, which because of the large number of atomic coordinates involved would be intractable to standard all-atom molecular dynamics approaches. While Arkhipov *et al.* [33], employing a unique special-purpose supercomputer, were able to perform all-atom simulations of a near complete dimeric EGFR structure of ∼5 µsec in duration, the structural model used in this investigation included no CT domain sequence elements and thus the self-phosphorylation reaction was again not illuminated. In this work, using a coarse-grained model of the full-length dimeric EGFR, we were able to observe over 1000 P-site binding events occurring over a total simulated time approaching 200 µsec (Figs. 4, 9 and 10), in a set of simulations that even with a coarse-grained model required nearly 150 days of computation.

The numbers of catalytic site encounters for the various P-sites of the EGFR CT domain occurring in our simulations should be related to the relative levels of P-site phosphorylation seen in biochemical experiments. We recognize that the frequencies of P-site binding seen in our simulations would not be exact predictors of the relative rates of P-site self-phosphorylation, given that they relate only to the substrate binding event and do not take into account the rate of the subsequent catalytic phospho-transfer reaction (*k*_cat_), which is presumably dependent upon the identity of the bound P-site. How the rates of phosphorylation of different P-sites sequences when bound in the catalytic site vary should be reflected in the steady state kinetics parameters reported by Fan *et al.* [8], who evaluated both *K*_M_ and *k*_cat_ for several 17-amino acid P-site-derived peptide substrates of the EGFR kinase (see Table 1). We propose that the relative rates of P-site self-phosphorylation (*v*_phos_) as would be determined biochemically can be predicted from the results of our P-site binding simulations by use of the relation
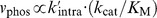
where *k′*_intra_ is the relative frequency of binding site interactions for a given P-site as determined in our simulations performed without CT domain electrostatic interactions and *k*_cat_ and *K*_M_ are the catalytic constants for the phosphorylation of the corresponding P-site-derived peptide [8] (see *Supporting Information*, Text S1). Predicted values of *v*_phos_ for those P-sites for which the steady state kinetic parameters of the corresponding peptides are available are given in Table 1, along with a qualitative characterization of the P-sites as major or minor sites of phosphorylation as based on a survey of the literature. P-sites-992, -1068, -1148 and -1173 had the highest *v*_phos_ values, and thus would be predicted to be efficiently phosphorylated, in agreement with the latter three being identified as major sites of phosphorylation. The low value of *v*_phos_ for P-site-1086, in part a reflection of its very low *k*_cat_*/K*_M_ ratio, is not consistent with its prior identification as a major site of phosphorylation. Conversely, while this analysis predicts that P-site-992 would be the most efficiently phosphorylated, it has not been considered a major site of EGFR phosphorylation. It should be recognized that our characterization of major and minor sites of EGFR phosphorylation was based upon the mainly *qualitative* biochemical analyses summarized in Table 2, including early phosphorylation site peptide mapping studies. While quantitative analyses have been more recently performed [34], [35], these have investigated EGFR sites phosphorylated in the context of live cells, wherein the levels of P-site phosphorylation are almost certainly modulated by the presence of P-site-binding proteins and protein phosphatases. Indeed, P-site-992 of the EGFR is an optimal substrate for the protein tyrosine phosphatase PTP-1B [36], which has been shown to dephosphorylate the EGFR in the cellular context [37]. Nonetheless, one quantitative study did identify P-site-992 as a major site of EGFR phosphorylation [34]. While the frequencies of P-site binding observed in our studies are thus generally consistent with available phosphorylation data, a more extensive analysis of the predictive capacity of our simulation strategy will require a quantitative analysis of *in vitro* EGFR phosphorylation under controlled conditions, experimentation that we are now initiating.

Awaiting a quantitative assessment of the results of our simulations, we note several intriguing particulars therein. Whereas there was overall a statistically significant increased propensity for *cis* versus *trans* phosphorylation, there was no significant difference when P-site-992 binding events were excluded from the analysis. Thus, while it is generally assumed that self-phosphorylation of the EGFR and other receptor PTKs occurs *in trans*, a belief promulgated by the observation that EGFR dimerization is required for PTK activation (e.g. [38]) and direct demonstrations of the phosphorylation of kinase-deficient PTK mutants by their wild-type counterparts (e.g. [39], see also [40]), our results indicate that *cis* and *trans* phosphorylation, at least in the case of the EGFR, should occur with a similar efficiency. This issue is particularly relevant in the context of the larger HER/ErbB family of receptors, in which the kinase-impaired HER3 receptor functions only upon its heterodimerization with the kinase-active EGFR, HER2 or HER4 receptor. Thus, while HER3 must in an EGFR/HER3 or HER2/HER3 heterodimer be phosphorylated primarily *in trans* [41], its partner EGFR or HER2 might still be phosphorylated *in cis* in such heterodimers, adding to the diversity of their downstream signaling. It should be noted that although HER2 is known to be phosphorylated in the context of HER2/HER3 heterodimers, this has also been attributed to the formation of higher-order receptor oligomers [42], [43].

On the other hand, the observation that P-site-992 interacted with the catalytic site exclusively *in cis* (see Fig. 4) is particularly provocative. First, this observation highlights the potential importance of topologic factors on EGFR signaling functions, such as would be difficult to investigate by methodologies other than that described herein. Second, this finding is again relevant in the context of heterodimeric HER/ErbB family receptors. With the kinase-proximal CT domain sequence up to and including P-site-992 being strongly conserved in the EGFR, HER2 and HER4 receptors (see Table 3), the possibility that the homologous P-sites within HER2 and HER4 are phosphorylated exclusively *in cis* appears strong. It is noteworthy that P-site-992 is not conserved in the HER3 C-terminal sequence. As HER3 possesses an impaired intrinsic kinase activity, this P-site if present would likely not be phosphorylated even in the context of heterodimeric receptors, and hence could have no canonical signaling function. This might explain its loss with the divergent evolution of HER/ErbB family members. Numerous related questions about receptor phosphorylation occurring in the context of homo- and hetero-dimeric HER/ErbB family receptors could be addressed by the simulation methodology described herein. For example, a recent study indicates that significant differences in EGFR phosphorylation site usage might underlie the higher potential for cancerous transformation of EGFR/HER2 heterodimers versus EGFR homodimers [44]. Are such differences in phosphorylation due to differing catalytic site selectivities among HER family receptors, or might they be attributed to topological factors that would differently impact self-phosphorylation occurring in hetero- versus homo-dimeric receptor forms?

Our discovery that the *in cis* phosphorylation of P-site-922 of the receiver monomer is facilitated by the presence of a cleft between the N-terminal and C-terminal lobes of the PTK domain (see Fig. 8) also suggests that the short CT-domain sequence appending P-site-922 to the kinase core is an element of a conserved mechanism for efficient intramolecular self-phosphorylation. While the presence of this cleft enables the *in cis* interaction of P-site-992 with the catalytic site, this does not necessarily involve an intimate binding interaction between the kinase-proximal CT domain sequence and PTK domain residues within the cleft. Thus, the threading of this acidic CT domain sequence through the cleft appears not to be stabilized by electrostatic interactions with complementary charged residues therein, consistent with the *in cis* catalytic site interactions of P-site-992 being markedly enhanced in those simulations in which CT domain charges were removed from the model (compare Figs. 4 and 9). Possibly, the P-site-992-catalytic site interaction facilitated by the cleft is necessarily transient in nature, to allow the phosphorylation of substrates other than P-site-992. This might explain why the kinase-proximal CT domain sequence is not seen to be localized within this cleft in any available crystallographic structure of the PTK domain. We note that P-site-992 is of particular biologic significance and the subject of much study. P-site-992 is involved in intracellular calcium signaling [45] and the induction of membrane ruffling in response to EGF [46] (apparently via its recruitment of signaling effectors including phospholipase C and Src), and is a negative regulator of the transforming activity of the EGFR-derived *v-ErbB* oncogene *in vivo* [47]. That phosphorylated P-site-992 is an optimal substrate for the protein tyrosine phosphatase PTP-1B [36], [37] suggests that the control of its phosphorylation level in cells is crucial.

Related to the issue of *cis* versus *trans* P-site-catalytic site interactions is our observation in some simulations that a productive interaction of a P-site with the catalytic site in the receiver monomer was delayed by a nonproductive interaction of a P-site(s) with the active site of the activator (see Fig. S2 and Videos S1, S2, S3 in *Supporting Information*). Thus, if a P-site of either CT domain interacted with the active site of the activator (located on the face of the PTK domain dimer opposing that of the catalytic site), it tended to preclude the interaction of other P-sites in the same CT domain with the true catalytic site. In some cases, a productive P-site/catalytic site binding event was preceded by several nonproductive P-site binding events involving the active site of the activator molecule (see *Supporting Information*, Fig. S2). How significantly such competition between alternative active sites impacted the observed frequencies of catalytic site binding events was not examined, and appears to be an unexplored issue regarding the asymmetric dimer allosteric activation mechanism [4]. Such active site competition might be particularly relevant in the case of HER3-containing receptor heterodimers in which the HER3 receptor would provide little if any intrinsic kinase activity but would provide an active site capable of binding ATP [48] and likely also the P-site substrates in the CT domain of its heterodimeric receptor partner.

We here also investigated the phenomenon of progressive multi-site self-phosphorylation of the EGFR (see Fig. 10). In examining the effects of phosphorylation of one P-site on subsequent P-site binding events, we found that mimicking the phosphorylation of one P-site (specifically P-site-992 of the receiver molecule) dramatically enhanced the frequency of binding of a P-site adjacent in sequence (specifically P-site-1045 of the receiver). The physical bases for such apparent processivity in phosphorylation could include the fact that the binding of one P-site to the catalytic site places those P-sites closest in sequence within a smaller distance of the catalytic site and, as we observed in several simulations, the possibility that the exchange of one catalytic site-bound P-site for another can occur by a sliding or translation of the CT domain through the catalytic cleft or by a physical displacement of one bound P-site by another adjacent in sequence (see *Supporting Information*, Fig. S2 and Video S3). Evaluating the impact of accumulated P-site phosphorylation on subsequent P-site phosphorylation events, such that one could predict the extent to which each P-site is phosphorylated when phosphorylation stoichiometries are high, would require a much longer series of simulations in which the step-wise phosphorylation of several P-sites was modeled. Because such accumulated P-site phosphorylation in the live cell would ultimately be opposed by the action of protein phosphatases and modulated by P-site binding proteins, the results of such modeling might not be predictive of the levels of P-site phosphorylation seen in analyses of EGFR phosphorylation in cultured cells or tissues.

Although our coarse-grained modeling of the EGFR self-phosphorylation reaction was necessitated by the large numbers of atoms involved and the limitations of the computational machinery available, we must recognize some limitations of our simulations. In the model employed, amino acid residues were represented by pseudo-atoms centered on the C_α_ atoms of the receptor polypeptides, and physical interactions between residues, including P-site/active site interactions, were treated with short-range attractive potentials between pseudo-atoms representing native contacts (residues pairs in close proximity in the original EGFR structures or in our active site-docked P-site structures) and with or without classical electrostatic interactions in the case of formally charged residues. Obviously, this model might not accurately recapitulate the atomistic steric interactions involved in active site substrate recognition and only approximates the sampling of conformational states of the CT domain polypeptide that occurs in the course of its movement. In terms of the former issue, we attempted to reintroduce those elements of substrate recognition lost in our modeling and the P-site-dependence of the rate of the catalytic phospho-transfer reaction by a simple theoretical treatment that takes advantage of published steady state catalytic constants for P-site phosphorylation. Regarding the latter issue, we must concede that the CT domain conformational sampling occurring in our simulations is an approximation of that occurring in reality, and the conclusions we make concerning the relative propensities of individual P-sites for active site binding are therefore tentative. We do consider that our simulations have identified possible new paradigms with regard to EGFR/HER family receptor signaling that might not otherwise have been discovered. These include the possibilities that *cis* and *trans* self-phosphorylation within dimeric receptors might occur with similar efficiencies and that the *in cis* interaction of P-site-992 with the catalytic site might be facilitated by a substrate binding cleft and could represent a unique evolutionarily conserved mechanism for the self-phosphorylation of this crucial regulatory P-site.

In summary, the investigation by simulation of the first step of the EGFR self-phosphorylation reaction we present here provides significant new insights into the mechanism of self-phosphorylation, and raises several intriguing questions about this mechanism in the contexts of the larger HER/ErbB receptor family and growth factor receptor PTKs in general. Issues that remain to be explored include the selectivity of P-site phosphorylation in heterodimeric receptors, such as in the highly transforming HER2/HER3 coreceptor combination that drives breast cancer progression [49], or how the binding of signaling effectors to phosphorylated P-sites as occurs in the cellular context would alter subsequent P-site phosphorylation. Such questions could be address by straightforward applications of the simulation methodology described herein. In predicting P-site phosphorylation rates from the results of our P-site-catalytic site binding simulations, the known catalytic efficiencies of P-site-derived peptides as derived from steady state kinetic studies were used to introduce elements of atomic-level molecular recognition that were lost in the coarse-grained modeling of EGFR structure. Further methodological developments might include a hybridized procedure in which large-scale movements of EGFR structural domains are treated by a coarse-grained approach and those associated with substrate recognition and catalysis by more refined atomistic methods.

## Materials and Methods

### Structural modeling of the active dimeric EGFR

A structure of the full-length active dimeric EGFR was created from known structures of the dimeric extracellular domain (3NJP) [17] and individual active (2GS6) and inactive (2GS7) conformation PTK domain structures [4] together assembled in an asymmetric dimer structure. (We use herein the amino acid numbering of residues in the mature EGFR molecule, i.e. not counting residues in the 24-residue signal peptide.) It is important to note that while the asymmetric PTK dimer model is reasonably well established, there exists no crystallographic structure for the asymmetric dimer, as the kinase domain apparently crystallizes in lattices of exclusively active or inactive conformation molecules [4]. Adjacent PTK domains in the active conformation crystal lattices do assume “receiver” and “activator” orientations, which led to the asymmetric PTK dimer model. Thus, in this work, the apposition of active (receiver) and inactive (activator) conformation PTK domains in an asymmetric dimer structure was effected by superpositioning an inactive conformation kinase structure (2GS7, residues 679 to 959) upon the activator-orientated kinase (residue 669 to 967) in a kinase dimer generated from the active-conformation crystal structure 2GS6 by a symmetry operation. In this superpositioning, performed with the aid of the Swiss PBD program [50], only those residues in the C-terminal lobe of the activator kinase near its interface with the N-terminal lobe of the receiver kinase (residues 904 to 955) were aligned. Next, because a related EGFR kinase domain structure (3GOP) from an active conformation-like lattice showed an additional structured portion of the intracellular JM sequence that was demonstrated to be important in stabilizing the asymmetric dimer [3], [51], the structure of this so-called “juxtamembrane latch” in 3GOP (residues 655 to 676) was appended to the structure of the active conformation kinase (residues 677 to 967) in our asymmetric dimer structure, after superpositioning of a common structural element of both structures (residues 675 to 693). Then, as residues 655 to 664 of the JM sequence are believed to assume an α-helical conformation in both activator and receiver kinases [3] but are not included in the inactive conformation kinase structure (2GS7) used for the activator in our symmetric dimer model, we positioned a copy of this short helical structure from 3GOP near the activator kinase N-terminus, such that it might be joined to the activator kinase by structural modeling of the intervening residues 665 to 678 (see below).

Because we wished to have a complete EGFR dimer model with structurally identical extracellular ligand binding domains, versus the nearly identical extracellular domains seen in the crystal structure 3NJP, we superposed a copy of molecule A of the 3NJP dimer structure upon molecule B in the original structure by aligning selected residues in the nearly symmetric contact interface between molecules A and B (residues 191 to 308 and 573 to 614). Thus, we constructed a nearly symmetrical EGFR extracellular domain dimer composed of structurally identical extracellular domains (residues 1 to 614) each with a bound EGF molecule, specifically residues 5 to 51 of the 53-residue EGF sequence that are ordered in the structure 3NJP. Subsequently, two transmembrane domain structures (residues 622–644) were modeled as α-helices by use of Swiss PDB, and remaining structural elements of the dimeric EGFR holoreceptor were modeled as follows.

The several structural elements described above [a dimer of identical EGFR extracellular domains (residues 1 to 614) with bound EGF molecules, two identical transmembrane helical segments (residues 622–644), an asymmetric dimer of active (residues 655 to 967) and inactive (residues 679 to 959) conformation PTK domains, and a short JM domain helical segment (residues 655 to 664) that would be included in the modeled JM domain of the inactive conformation activator molecule] were manually reoriented with respect to each other in the Swiss PDB viewer such that they might be connected by modeling the missing structural elements, specifically residues 615 to 621 of the two extracellular JM domains, residues 645 to 654 of the intracellular JM sequence proximal to the active conformation kinase domain, residues 645 to 654 and 665 to 678 of the intracellular JM sequence that connect the TM domain, JM domain helical segment, and inactive conformation kinase structures, and lastly, the two CT domains of the active (residues 968 to 1186) and inactive (960 to 1186) conformation kinases. (The few disordered residues at the extreme N- and C-termini of the bound EGF molecules were also modeled, as were short disordered sequences within each of the PTK domain structures.) The several segments connecting known structures were structurally modeled with the Loopy algorithm [18]. The various N- and C-terminal sequence extensions were modeled with an in-house program that sequentially builds up such structures by replacing the terminal amino acid residue of the chain being extended with a dipeptide structure of the appropriate sequence that is randomly chosen from a library of allowable dipeptide structures (all those found in the PBD structure database), aligns the backbone conformation of the first dipeptide residue with that of the terminal amino acid replaced, and checks that the structure of neither dipeptide residue clashes with other structural elements. The result was a structural model of a dimer of full-length EGFR molecules, each with bound EGF and an ∼225 amino acid CT domain in a random conformation (Fig. 1).

As no available active conformation EGFR kinase structure contained a nucleotide substrate complex of ATP (or the analog AMPPNP) and two bound Mg^2+^ cations, as would be presumed to be the relevant substrate for the self-phosphorylation reaction, we docked the nucleotide substrate complex (AMPPNP with two chelated Mg^2+^ cations) from a structure of the active insulin receptor PTK domain with bound nucleotide and peptide substrates (1IR3) [52] into the active site of each kinase monomer. In the case of the active conformation receiver kinase, this was done by aligning the 1IR3 and 2GS6-derived kinase domain structures, which placed the 1IR3 nucleotide substrate complex into the EGFR active site without clashes and positioned appropriately with respect to critical catalytic residues (Fig. 2). In the case of the inactive conformation activator kinase, the conformation of which was significantly different from that of 1IR3, we aligned the AMPPNP of the bound AMPPNP^.^2Mg^2+^ complex in 1IR3 with the AMPPNP in the 2GS7-derived structure, which again appropriately placed the substrate complex in the active site.

### Structural modeling of P-site interactions with the EGFR PTK active site

Each CT domain in the active receptor dimer contains eight candidate P-sites that potentially interact with the active site of the receiver molecule (referred to herein as the catalytic site) and become phosphorylated. Our simulation of the self-phosphorylation reaction therefore required the generation of structural models descriptive of the interaction of each P-site with the EGFR catalytic site. Because in most cases the primary determinants of protein kinase substrate specificity are located within four residues on either side of the target residue [53] and because we intended to monitor when the target tyrosine residue of any one P-site would be bound in the catalytic site and positioned appropriately to promote its phosphorylation, we considered that modeling of the catalytic site interactions of relatively short P-site sequences would be sufficient for our purposes. Thus, we created structural models of individual nine-amino acid P-site peptides (comprising EGFR residues *N*-4 to *N*+4, where residue *N* is the P-site tyrosine, see Table 1) interacting with the active conformation EGFR kinase. As no suitable structure of the EGFR kinase domain with a bound peptide substrate was available, we exploited the structure of the active insulin receptor kinase with a bound IRS-2-derived peptide substrate (3BU3) [54]. Upon aligning the insulin receptor kinase domain in this structure with that of the active conformation kinase domain in our dimeric EGFR structure, the nine relevant amino acids of the IRS-2 peptide surrounding its target tyrosine were seen to be well accommodated in the EGFR kinase catalytic cleft, and a hybrid structure of the EGFR PTK domain with a bound IRS-2 peptide could be generated. This structure was then used to model with the Swiss PDB program the conformation and EGFR catalytic site interactions of each of the eight, nine-amino acid P-site peptide sequences of the EGFR CT domain. For the purpose of generating a coarse-grained EGFR structural and energetic model that incorporated physical interactions between the P-site sequences and the EGFR active site (see below), the P-site peptides in each of the eight docked structures were linked to the EGFR kinase sequence by a randomly structured CT domain extension (residues 968 to 987 of the EGFR), generated by application of Loopy [18] (see Fig. 8). In each model structure, the central tyrosine residue of the P-site peptide substrate assumed a conformation identical to that of the docked IRS-2 peptide, thus with its tyrosine hydroxyl in close proximity of the EGFR catalytic aspartate residue (Asp-813) and the γ-phosphate of the bound AMPPMP substrate (see Fig. 2). The interaction of individual P-site peptides with the kinase active site involved close interactions with active site residues that appeared to be more electrostatically favorable in the case of some P-site peptides.

### A coarse-grained structural and energetic model of the active dimeric EGFR

Given an all-atom model of the dimeric EGFR and eight models of the EGFR kinase domain each with a distinct P-site peptide docked in the active site, a coarse-grained structural and energetic model was generated using the approach of Gō [20] as recently implemented [25], using software generously made available by Dr. Adrian Elcock (University of Iowa). Briefly, each amino acid residue of the peptide components was represented by a pseudo-atom centered on its C_α_ atom with bonds formed between pseudo-atoms of adjacent residues. Models of the bound AMPPNP^.^2Mg^2+^ complexes comprised pseudo-atoms centered on atoms C2, C6 and N9 of the adenine moiety, C4′ of the deoxyribose, P1, P2 and P3 of the α-, β-, and γ-phosphates, and each of the two Mg atoms, with bonds connecting every pseudo-atom pair. The energetic model was the summation of (1) standard molecular mechanics potential functions to treat interactions between bonded pseudo-atoms, (2) Gō-inspired potential functions to model short-range attractive interactions between those non-bonded pseudo-atoms representing native contacts, (3) short-range repulsive potentials to treat interactions between all other non-bonded pseudo-atoms, and (4) electrostatic potential terms relating to all charged residue/atom pairs (cf. [25]). The values assigned to the various parameters in the energetic model as indicated below were those shown to be optimal for accurate coarse-grained modeling of protein folding thermodynamics and protein diffusion [25], [55].

In the energetic model, the interactions between bonded pseudo-atoms were described by
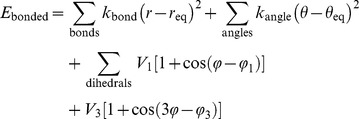
where *r*, *θ*, and *φ* are the bond distances, bond angles, and bond dihedral angles, respectively, *r*_eq_ and *θ*_eq_ are the corresponding bond distances and angles in the initial structure, and *φ*_1_ and *φ*_3_ are phase angles defining the position of the energy maxima of the cosine terms. (The multiply interconnected pseudo-atoms of the AMPPNP^.^2Mg^2+^ substrate complexes were assigned only harmonic bond distance and bond angle energy terms.) The force constants *k*_bond_ and *k*_angle_ were set to 20 kcal/mol/Å^2^ and 10 kcal/mol/rad^2^, respectively, and the energy maxima *V*_1_ and *V*_3_ for the dihedral terms were set to 0.5 and 0.25 kcal/mol, respectively.

The interactions between non-bonded pseudo-atoms (those separated by three or more residues) were modeled with additional short-range attractive potential functions
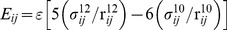
in the cases of those non-bonded pseudo-atoms *i* and *j* that represented native contacts. A residue was considered to form a native contact if any of its atoms were within a specified distance cut-off (here 5.5 Å) of any of another residue in the native structure, with exceptions as described below. Here the energy well depth *ε* was assigned a value of 0.6 kcal/mol, and *σ_ij_* and *r_ij_* are the distances separating the pseudo-atoms in the native and evolving structures, respectively. Non-bonded pseudo-atoms not representing native contacts were modeled with strictly repulsive terms:
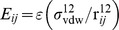
with *σ*_vdw_ set here at 4 Å and *ε* at 0.6 kcal/mol. In addition to these bonded and non-bonded energy terms, any pseudo-atom representing a residue (or atom in the AMPPNP^.^2Mg^2+^ substrate complex) with a significant partial charge at the simulation pH (here 7.6) was assigned that partial charge and allowed to interact with other such pseudo-atoms via additional Debye-Hückel electrostatic potential terms
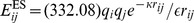
where *q_i_* and *q_j_* are the charges on pseudo-atoms *i* and *j*, *κ* is the ionic strength (set at 0.15 M), 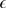 is the solvent dielectric constant (set at 78.4), and *r*_ij_ is again the distance between the pseudoatoms.

A membrane in which the dimeric receptor was constrained to diffuse laterally was modeled with two planar barriers normal to the *z*-axis (with which the EGFR TM domains were aligned) and separated by 33 Å, and additional terms in the energetic model that held the TM domain residues between the membrane planes and kept extracellular and intracellular EGFR structural elements from penetrating the membrane. Specifically, each pseudo-atom representing one of the TM residues 622 to 644 was subject to a restraining potential
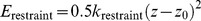
where *z*_0_ and *z* are the initial and evolving, respectively, displacements of the TM pseudo-atom along the *z*-axis and *k*_restraint_ is a force constant set at 5.0 kcal/mol/Å^2^. Also, extracellular pseudo-atoms (those of the receptor extracellular domains and bound EGF molecules) were subject to half-harmonic repulsive potential terms
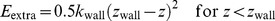

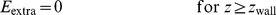
where *z* is the evolving *z*-coordinate of the extracellular pseudo-atom, *z*_wall_ indicates the position (16.5 Å) of the extracellular membrane, and *k*_wall_ is a force constant set again at 5.0 kcal/mol/Å^2^. Analogous repulsive potential terms applied to intracellular pseudo-atoms (those of the intracellular domains and the nucleotide substrate complexes).

The all-atom structural model of the dimeric EGFR from which our coarse-grained model was generated contained elements of known structure derived from published crystal structures, as well as some modeled structural elements (e.g. sequences in the extracellular and intracellular JM domains and the entirety of the two CT domains). Only those pseudo-atoms representing residues in elements of known structure were considered able to form native contacts and thus interact with other such non-bonded pseudo-atoms via short-range attractive versus purely repulsive potentials, which ensured that the folding of the structured domains remained intact during dynamic simulations. There were two exceptions. Firstly, those pseudo-atoms of the active site-bound AMPPNP^.^2Mg^2+^ complexes representing atoms within 5.5 Å of any atom of an active site residue in the all-atom model were considered to form native contacts and assigned short-range attractive potential terms corresponding to these interactions. Secondly, those residues of the eight different nine-amino P-site sequences that formed contacts with active site residues (or atoms of the AMPPNP^.^2Mg^2+^ substrate complex) when docked in the kinase active site were also considered to make native contacts (again a 5.5 Å cutoff was applied). In the case of each P-site peptide, the P-site tyrosine in the docked structure formed nine total native contacts with active site residues (in addition to three involving the γ-phosphate of the bound AMPPNP substrate), and the simultaneous formation of these nine contacts in the course of a simulation was used to define a stable P-site/catalytic site interaction. This was analogous to evaluating the extent of protein folding using a reaction coordinate or order parameter (e.g. *Q*) defined as the fraction of native contacts formed in the evolving system [21].

### Molecular simulations of EGFR P-site binding events

Given the above coarse-grained structural and energetic model, the motion of the dimeric EGFR structure was propagated using a Langevin dynamics algorithm developed by Winter and Geyer [19] and implemented with software provided by Dr. Adrian Elcock (University of Iowa). Simulations used an integration time step of 125 fsec. A list of nonbonded interactions was computed every 6 psec, short-range interactions (between pseudo-atoms within 12.5 Å) were computed every time step, and medium-range interactions (between pseudo-atoms greater than 12.5 Å and less than 25 Å apart) every 1 psec. No cutoff was applied to electrostatic interactions. The diffusion tensor descriptive of hydrodynamic interactions was calculated every 24 psec. Hydrodynamic radii of pseudo-atoms representing amino acid residues were set at 5.3 Å and those of the somewhat finer-grained AMPPNP^.^2Mg^2+^ substrate complex model set at 3.5 Å. In the course of P-site binding simulations, the number of P-site tyrosine/catalytic site native contacts formed was periodically determined, and the simulation terminated if all of the nine possible contacts were simultaneously formed in the case of any P-site. Simulations were performed in sets of five on separate Dell PowerEdge R815 computers, each with four AMD Opteron 6168 12-core processors. Snapshots of simulations were saved every 100 psec for analysis.

### Statistical analysis

A multinomial test was used to determine if differences in the frequencies of P-site binding were statistically significant.

### Molecular structure analysis and graphics

In structural modeling, homologous structural elements were aligned using the superpositioning functions of the Swiss PDB program [50]. All molecular structure representations were generated with VMD [56].

## Supporting Information

Figure S1 **Influence of initiating structures on the relative frequency of simulated P-site binding events.** P-site binding simulations were performed iteratively on five computers with initial structures randomly chosen from five different sets of one hundred structural models derived from five trajectories of 10 µsec randomizations of independent EGFR structures (see Fig. 3 and *Materials and Methods*). We assumed that CT domain conformational alterations occurring in the 100 nsec intervals of simulation time separating each consecutive structure in these sets would be sufficient to obviate any potential influence of the choice of initiating structures on the outcome of the simulations. To test this assumption, we examined whether the identity of the P-site interacting in a given simulation was correlated with the trajectory time of the initial structure used in the simulation. Thus, for all pairs of simulations in which the same P-site interacted with the catalytic site, we determined the interval of simulation time (Δ*t*) separating the two initial structures used in the simulations, and made a histogram of the number of same-site binding events versus Δ*t*. Because the relative likelihood (*p*) that two times chosen randomly from a total time interval *T* (here 10 µsec) are different by a time Δ*t* decreases linearly with Δ*t* according to *p* = 2^.^(1−Δ*t*/*T*), we evaluated for comparison the frequency with which differing Δ*t* values would arise via a random selection of the same total number of pairs of time values. If simulations initiated with structures more closely spaced in trajectory time had an increased tendency to result in the binding of the same P-site, the observed frequency of same-site binding events (Simulated Events) with smaller Δ*t* values would be greater than the frequency with which such Δ*t* values would arise from randomly selecting pairs of times from within the interval *T* (Uncorrelated Events). The analysis showed that only for pairs of initial structures most closely spaced in trajectory time (<500 nsec) was there a significantly higher propensity for same-site binding events. This indicated that although the propensity for binding of a given P-site in a simulation was somewhat dependent upon the initiating structure, the diversity of the set of initiating structures sampled from the 10 µsec trajectories of the five independent structural randomizations would largely negate any dependence of the observed frequencies of interaction of differing P-sites upon the initiating structures.
(TIF)

Figure S2 **Potential competition of the non-catalytic active site with the true catalytic site in P-site binding.** In some simulations, the binding of a P-site to the catalytic site in receiver molecule appeared to be delayed by the binding of a P-site(s) to the alternative active site in the activator molecule, which restricted the access of the CT domain containing that P-site to the catalytic site (see also Videos S1, S2, S3). When P-site binding was especially delayed, there were often a number of nonproductive interactions of a P-site with the activator active site that preceded the binding of a P-site to the true catalytic site. Shown here are plots of P-site/activator active site distances for P-sites interacting nonproductively with the active site of the activator molecule over the course of one such simulation taken from those described in Fig. 4 and lasting a total of 2.7 µsec. Plotted are the distances between pseudoatoms representing the tyrosine residues of the indicated P-sites and Asp-813B (with A and B indicating sites in the receiver and activator, respectively) in the active site of the activator, with a binding interaction indicated when the distance remained stable for a period at ∼12 Å, the distance of a closet approach. The simulation begins with the nonproductive binding of P-site-1101B, followed in succession by the binding of P-site-1086B, -1068B, -1086B, -1101A, -1068B, -1173B, and -1045B, and ending with the binding of P-site-1101B to the true catalytic site (not shown). Thus, in this simulation, eight nonproductive binding events preceded the binding of a P-site to the true catalytic site. Note that the exchange of bound P-sites sometimes involved two sites that were neighboring in sequence (e.g. P-sites-1068 and -1086), a possible mechanism for processivity in P-site phosphorylation (see Fig. 10 and discussion thereof). Note also that repeated P-site binding events were seen only in the case of the non-catalytic active site, because simulations were terminated when a P-site/catalytic site interaction occurred.
(TIF)

Video S1 **Representative P-site binding simulation showing competition between active sites.** Animation showing a trajectory from a representative P-site binding simulation in the first of three views (see also Videos S2 and S3). Pseudo-atoms of the receiver and activator receptor monomers are represented as beads colored in shades of pink and blue, respectively, with bound growth factor molecules in dark blue. In both monomers the catalytic Asp-813 residue is colored cyan and the tyrosines of all P-sites are colored green. The 1840 trajectory images represent 1 nsec intervals during the complete 1.8 µsec simulation. This first animation shows the motion of the dimeric EGFR from a view through the plane of the membrane. The simulation begins with P-site-1148A of the receiver monomer binding to the active site of activator (see close-up in Video S3, at ∼5 sec), which is followed by its displacement by P-site-1045A (at ∼30 sec), continues with the displacement of P-site-1045A by P-site-1068A (at ∼50 sec), and ends with the binding of P-site-992A to the catalytic site of the receiver (last frame, see also Video S2). The simulation suggests that the binding of P-sites to the active site of the activator monomer can delay the interaction of another P-site with the true catalytic site in the receiver monomer.
(MPG)

Video S2 **Representative P-site binding simulation showing competition between active sites: view of receiver catalytic site.** Close-up animation of the trajectory shown in Video S1 but with the pseudo atoms of the receiver PTK domain in each trajectory image aligned and shown in an orientation with the catalytic site of the receiver in view. The simulation begins with P-site-1148A of the receiver monomer binding to the active site of activator (at ∼5 sec, see also Video S3), which is followed by its displacement by P-site-1045A (at ∼30 sec), continues with the displacement of P-site-1045A by P-site-1068A (at ∼50 sec), and ends with the binding of P-site-992A to the catalytic site of the receiver (last frame).
(MPG)

Video S3 **Representative P-site binding simulation showing competition between active sites: view of activator active site.** Close-up animation of the trajectory shown in Video S2 but in an orientation with the active site of the activator in view. The simulation begins with P-site-1148A of the receiver monomer binding to the active site of activator (at ∼5 sec), which is followed by its displacement by P-site-1045A (at ∼30 sec), continues with the displacement of P-site-1045A by P-site-1068A (at ∼50 sec), and ends with the binding of P-site-992A to the catalytic site of the receiver (last frame, see also Video S2).
(MPG)

Text S1 **Interpretation of simulated P-site binding event frequencies.** A theoretical digression on the prediction of P-site phosphorylation rates by use of the frequencies of P-site binding observed in simulations and the steady state catalytic parameters for phosphorylation of P-site-derived peptides (citing references [60], [61]).
(PDF)
