# Supplementary figures and images for: Coarse-Grained Molecular Simulation of Epidermal Growth Factor Receptor Protein Tyrosine Kinase Multi-Site Self-Phosphorylation
Source: PLoS Comput Biol. 2014 Jan 16;10(1):e1003435. doi: 10.1371/journal.pcbi.1003435 (PMC3894164; doi:10.1371/journal.pcbi.1003435)

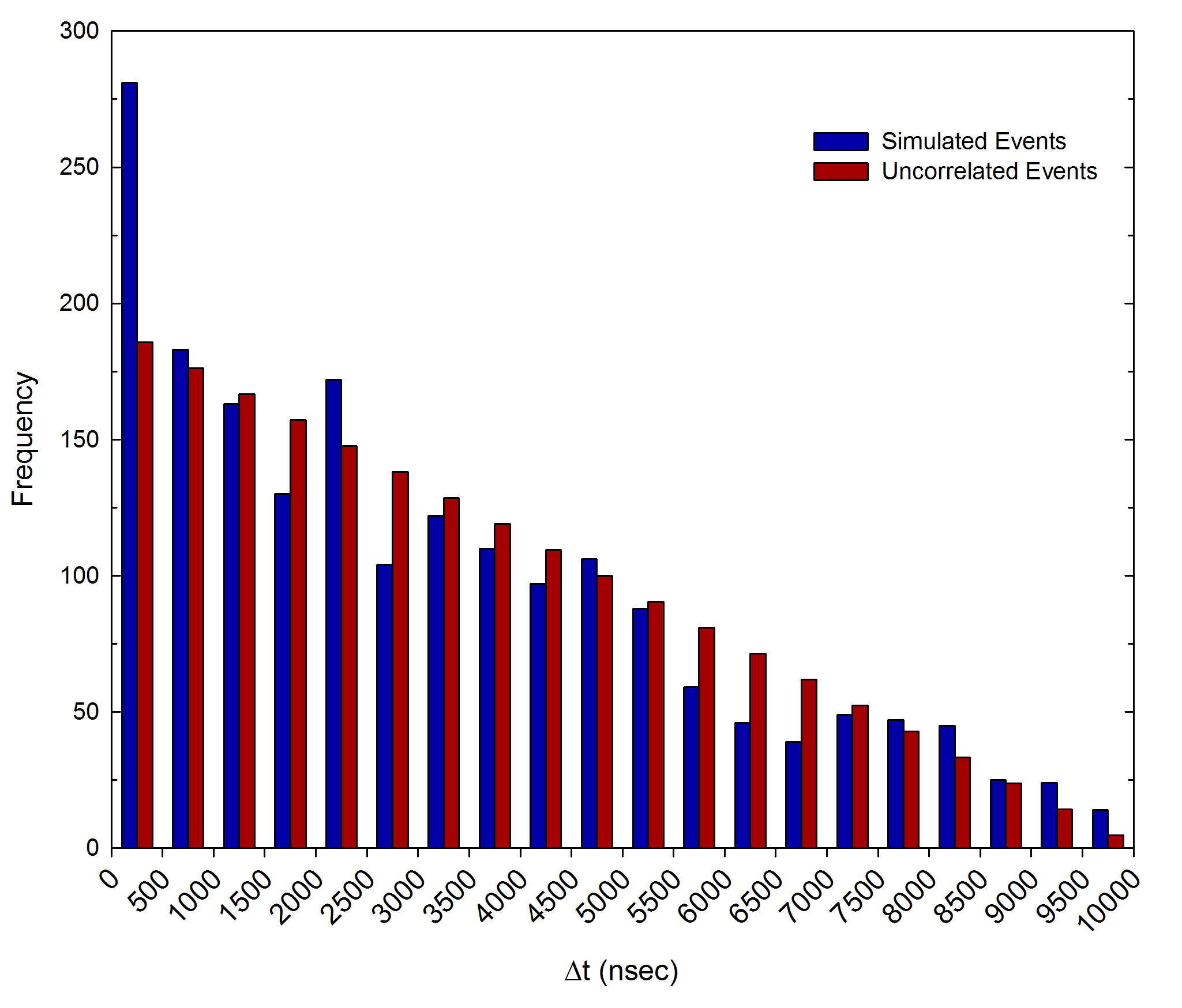

Supplement: Figure S1 — Influence of initiating structures on the relative frequency of simulated P-site binding events. P-site binding simulations were performed iteratively on five computers with initial structures randomly chosen from five different sets of one hundred structural models derived from five trajectories of 10 µsec randomizations of independent EGFR structures (see Fig. 3 and Materials and Methods). We assumed that CT domain conformational alterations occurring in the 100 nsec intervals of simulation time separating each consecutive structure in these sets would be sufficient to obviate any potential influence of the choice of initiating structures on the outcome of the simulations. To test this assumption, we examined whether the identity of the P-site interacting in a given simulation was correlated with the trajectory time of the initial structure used in the simulation. Thus, for all pairs of simulations in which the same P-site interacted with the catalytic site, we determined the interval of simulation time (Δt) separating the two initial structures used in the simulations, and made a histogram of the number of same-site binding events versus Δt. Because the relative likelihood (p) that two times chosen randomly from a total time interval T (here 10 µsec) are different by a time Δt decreases linearly with Δt according to p = 2.(1−Δt/T), we evaluated for comparison the frequency with which differing Δt values would arise via a random selection of the same total number of pairs of time values. If simulations initiated with structures more closely spaced in trajectory time had an increased tendency to result in the binding of the same P-site, the observed frequency of same-site binding events (Simulated Events) with smaller Δt values would be greater than the frequency with which such Δt values would arise from randomly selecting pairs of times from within the interval T (Uncorrelated Events). The analysis showed that only for pairs of initial structures most closely spa [file pcbi.1003435.s001.tif]

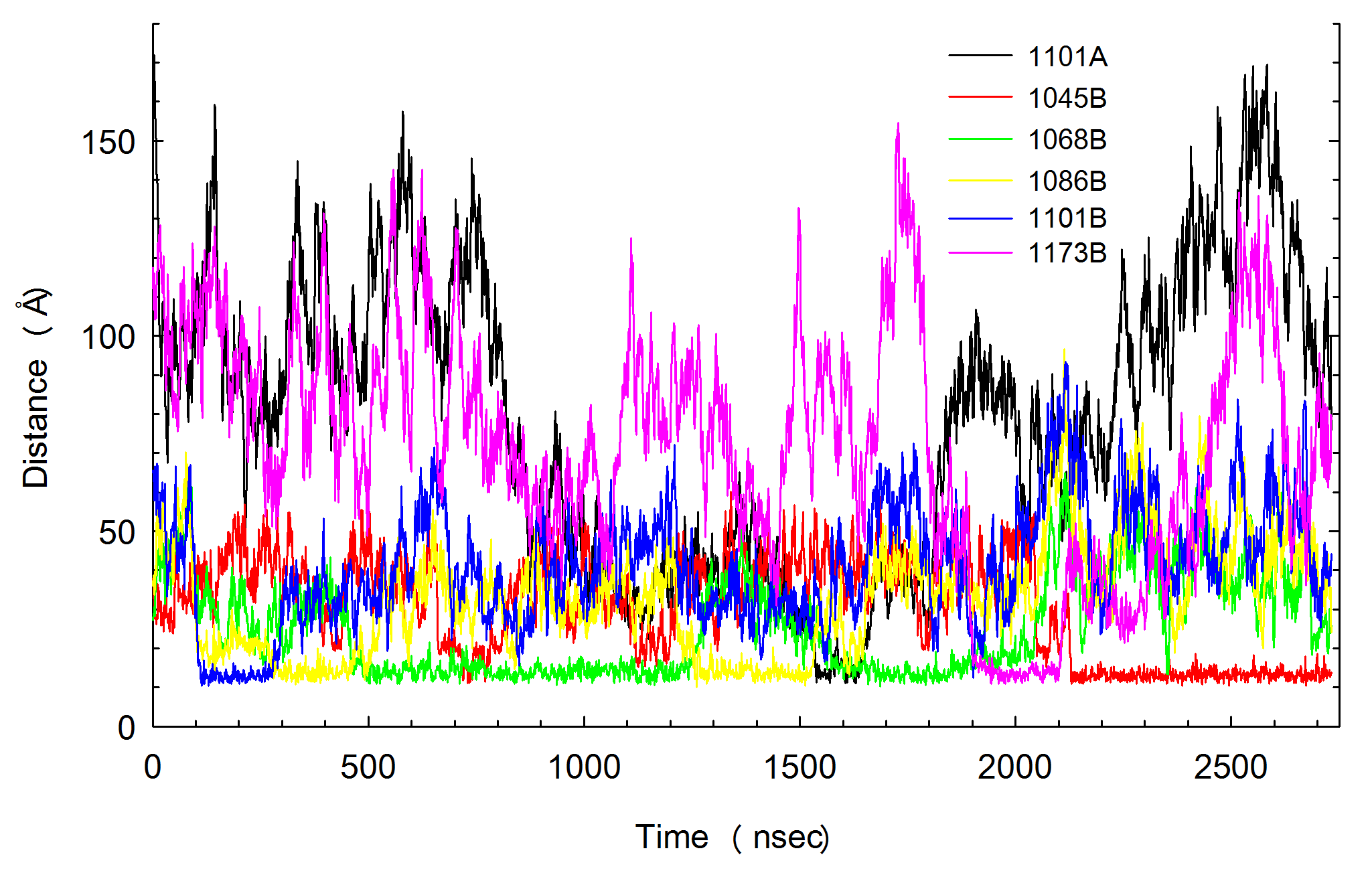

Supplement: Figure S2 — Potential competition of the non-catalytic active site with the true catalytic site in P-site binding. In some simulations, the binding of a P-site to the catalytic site in receiver molecule appeared to be delayed by the binding of a P-site(s) to the alternative active site in the activator molecule, which restricted the access of the CT domain containing that P-site to the catalytic site (see also Videos S1, S2, S3). When P-site binding was especially delayed, there were often a number of nonproductive interactions of a P-site with the activator active site that preceded the binding of a P-site to the true catalytic site. Shown here are plots of P-site/activator active site distances for P-sites interacting nonproductively with the active site of the activator molecule over the course of one such simulation taken from those described in Fig. 4 and lasting a total of 2.7 µsec. Plotted are the distances between pseudoatoms representing the tyrosine residues of the indicated P-sites and Asp-813B (with A and B indicating sites in the receiver and activator, respectively) in the active site of the activator, with a binding interaction indicated when the distance remained stable for a period at ∼12 Å, the distance of a closet approach. The simulation begins with the nonproductive binding of P-site-1101B, followed in succession by the binding of P-site-1086B, -1068B, -1086B, -1101A, -1068B, -1173B, and -1045B, and ending with the binding of P-site-1101B to the true catalytic site (not shown). Thus, in this simulation, eight nonproductive binding events preceded the binding of a P-site to the true catalytic site. Note that the exchange of bound P-sites sometimes involved two sites that were neighboring in sequence (e.g. P-sites-1068 and -1086), a possible mechanism for processivity in P-site phosphorylation (see Fig. 10 and discussion thereof). Note also that repeated P-site binding events were seen only in the case of the non-catalytic active site, because simulations wer [file pcbi.1003435.s002.tif]
