# Supplementary material for: Coarse-Grained Molecular Simulation of Epidermal Growth Factor Receptor Protein Tyrosine Kinase Multi-Site Self-Phosphorylation
Source: PLoS Comput Biol. 2014 Jan 16;10(1):e1003435. doi: 10.1371/journal.pcbi.1003435 (PMC3894164; doi:10.1371/journal.pcbi.1003435)
Supplement: Text S1 — Interpretation of simulated P-site binding event frequencies. A theoretical digression on the prediction of P-site phosphorylation rates by use of the frequencies of P-site binding observed in simulations and the steady state catalytic parameters for phosphorylation of P-site-derived peptides (citing references [60], [61]). (PDF) [file pcbi.1003435.s006.pdf]

Supporting Information

Text S1.

Interpretation of simulated P-site binding event frequencies

The frequencies of catalytic site binding for the various P-sites occurring in our coarse-grained molecular simulations (see Figs. 4 and 9) should be related to the levels of P-site phosphorylation that would be seen in biochemical assays of receptor self-phosphorylation. Considering a proposed rapid equilibrium model of EGFR catalysis [60] in which the phospho-transfer reaction is preceded by the reversible binding of the peptide substrate to the ATP-occupied catalytic site (as would be predicted in the presence of saturating ATP concentrations), the observed frequency of binding for a given P-site *i* (*i* = 1-16, indicating each of the eight P-sites of the two molecules in the EGFR dimer) as simulated in this study would reflect the relative rate of the P-site-catalytic site association reaction ($k'_{1,i}$) (with *'* indicating this is not a second-order rate constant but first-order as would characterize an intramolecular reaction). The relative initial velocity of phosphorylation of P-site *i* ($v_{\mathrm{phos},i}$) as determined biochemically would be estimated by multiplying these frequencies by the rate constant $k_{\mathrm{cat},i}$ for the phospho-transfer reaction and dividing by the off-rate $k_{-1,i}$ for the P-site-catalytic site binding event (values presumably different for each P-site):

$$v_{\mathrm{phos},i} = k'_{1,i} \cdot k_{\mathrm{cat},i} / k_{-1,i} \tag{1}$$

We note that there is a closely related expression (assuming again rapid equilibrium) for the catalytic efficiencies for P-site-derived synthetic peptide phosphorylation by the EGFR, such as seen in the steady state kinetics experiments of Fan *et al.* [8] (see Table 1):

$$k_{\mathrm{cat},i} / K_{\mathrm{M},i} = k_{1,i} \cdot k_{\mathrm{cat},i} / k_{-1,i} \tag{2}$$

except that the constants $k_{1,i}$, instead of containing information about the constrained diffusion of P-sites in an *intramolecular* association reaction as do the $k'_{1,i}$ values above, characterize the second-order catalytic site association reaction for the P-site-derived synthetic peptides.

Combining these expressions 1 and 2 yields:

$$v_{\mathrm{phos},i} = \left(k'_{1,i} / k_{1,i}\right) \cdot \left(k_{\mathrm{cat},i} / K_{\mathrm{M},i}\right) \tag{3}$$

We might assume the $k'_{1,i}$ values for the self-phosphorylation reaction contain information about the relative rates of *intramolecular* diffusional encounters for the individual P-sites ($k'_{\mathrm{intra},i}$) and information about the physical interaction of the individual P-sites with the active site (electrostatic attraction/repulsion, steric effects, etc.) ($f_{\mathrm{phys},i}$) such that

$$k'_{1,i} = f_{\mathrm{phys},i} \cdot k'_{\mathrm{intra},i} \tag{4}$$

(This is akin to a using modified Smoluchowski equation to treat diffusional and steric factors for an enzyme substrate association reaction, cf. [61].) Similarly, the $k_{1,i}$ values for the synthetic peptide phosphorylation experiments could be assumed to depend upon the rate of *intermolecular* diffusional encounters ($k_{\mathrm{inter},i}$) and the same physical information ($f_{\mathrm{phys},i}$) or

$$k_{1,i} = f_{\mathrm{phys},i} \cdot k_{\mathrm{inter},i} \tag{5}$$

Then it would follow, by taking the ratio of 4 and 5, that

$$k'_{1,i} / k_{1,i} = k'_{\mathrm{intra},i} / k_{\mathrm{inter},i} \tag{6}$$

and, by combining 3 and 6,

$$v_{\mathrm{phos},i} = \left(k'_{\mathrm{intra},i} / k_{\mathrm{inter},i}\right) \cdot \left(k_{\mathrm{cat},i} / K_{\mathrm{M},i}\right) \tag{7}$$

If our simulations approximate the relative rates of *intramolecular* diffusional encounters for the individual P-sites ($k'_{\mathrm{intra},i}$) in the absence of physical factors (electrostatic and steric interactions), as would our course-grained simulations performed in absence of CT domain charges (see Fig. 9), and if the rates of *intermolecular* diffusion $k_{\mathrm{inter},i}$ in the absence of physical factors are equivalent for synthetic peptides of the same size, then the *relative* rates of P-site self-phosphorylation would be obey the proportionality

$$v_{\mathrm{phos},i} \propto k'_{\mathrm{intra},i} \cdot \left(k_{\mathrm{cat},i} / K_{\mathrm{M},i}\right) \tag{8}$$

This is to say that the *relative* rates of P-site self-phosphorylation in biochemical experiments should be predicted by multiplying the catalytic efficiencies observed in peptide phosphorylation experiments by the relative frequencies of P-site binding seen in our simulations. Predicted relative rates of P-site self-phosphorylation (v_{phos}) were thus obtained using the results from simulations with no CT domain charges (Fig. 9, totals of c*is* and *trans* binding events) and the catalytic constants of Fan *et al.* [8], and are presented in Table 1.
